# Forest Structure in Low-Diversity Tropical Forests: A Study of Hawaiian Wet and Dry Forests

**DOI:** 10.1371/journal.pone.0103268

**Published:** 2014-08-27

**Authors:** Rebecca Ostertag, Faith Inman-Narahari, Susan Cordell, Christian P. Giardina, Lawren Sack

**Affiliations:** 1 Department of Biology, University of Hawai‘i at Hilo, Hilo, Hawai‘i, United States of America; 2 Department of Natural Resources and Environmental Management, University of Hawai‘i at Mānoa, Honolulu, Hawai‘i, United States of America; 3 Institute of Pacific Islands Forestry, Pacific Southwest Research Station, USDA Forest Service, Hilo, Hawai‘i, United States of America; 4 Department of Ecology and Evolutionary Biology, University of California Los Angeles, Los Angeles, California, United States of America; Cirad, France

## Abstract

The potential influence of diversity on ecosystem structure and function remains a topic of significant debate, especially for tropical forests where diversity can range widely. We used Center for Tropical Forest Science (CTFS) methodology to establish forest dynamics plots in montane wet forest and lowland dry forest on Hawai‘i Island. We compared the species diversity, tree density, basal area, biomass, and size class distributions between the two forest types. We then examined these variables across tropical forests within the CTFS network. Consistent with other island forests, the Hawai‘i forests were characterized by low species richness and very high relative dominance. The two Hawai‘i forests were floristically distinct, yet similar in species richness (15 vs. 21 species) and stem density (3078 vs. 3486/ha). While these forests were selected for their low invasive species cover relative to surrounding forests, both forests averaged 5–>50% invasive species cover; ongoing removal will be necessary to reduce or prevent competitive impacts, especially from woody species. The montane wet forest had much larger trees, resulting in eightfold higher basal area and above-ground biomass. Across the CTFS network, the Hawaiian montane wet forest was similar to other tropical forests with respect to diameter distributions, density, and aboveground biomass, while the Hawai‘i lowland dry forest was similar in density to tropical forests with much higher diversity. These findings suggest that forest structural variables can be similar across tropical forests independently of species richness. The inclusion of low-diversity Pacific Island forests in the CTFS network provides an ∼80-fold range in species richness (15–1182 species), six-fold variation in mean annual rainfall (835–5272 mm yr^−1^) and 1.8-fold variation in mean annual temperature (16.0–28.4°C). Thus, the Hawaiian forest plots expand the global forest plot network to enable testing of ecological theory for links among species diversity, environmental variation and ecosystem function.

## Introduction

High species richness is a hallmark of many tropical forests [1], [2]. Indeed, the latitudinal gradient and equatorial peak in plant diversity has attracted attention for centuries e.g., [1], [3], [4], [5]. Numerous studies have focused on the causes of high diversity in tropical forests [1], [6], [7], [8], [9], [10], and theories have been formulated to explain how species or functional diversity in turn affects ecosystem function [11], [12]. However, these linkages have rarely been tested, and not all tropical forests are diverse. For example, legume-dominated swamp forests, peat forests, pine savannas, and oceanic islands that are geographically isolated can have low to very low diversity [13], [14], [15], [16]. Such low-diversity forests are understudied, and there is no clear answer to the simple question of whether the structure of a low-diversity tropical forest would be expected to be similar to or different from that of a high-diversity tropical forest with comparable climate.

Indeed, the question of how forest structure—i.e., physiognomy, basal area, density, diameter size class distributions, biomass, and evenness—varies with species diversity is itself understudied, likely an effect of the paucity of studies of the structure of low-diversity tropical forests. Some have hypothesized that forest structure and species-richness might be related, if structure acts as a habitat scaffold or template that precedes and enables species assembly and diversity by providing an increased variety of habitat niches (e.g., nurse logs for seedlings, perches for birds that disperse seeds, climbing structures for vines [17], [18]. Alternatively, higher diversity may enhance forest structure, if more species correspond to a wider variety of size classes, strata, and crown architectures [17]. Both processes are not mutually exclusive and may operate simultaneously, creating a positive feedback cycle that would enhance diversity and influence various forest structural attributes. Recent efforts have examined some structural variables, such as latitudinal trends in height across forests e.g., [19], [20] and the effects of diversity and spatial scale on standing forest biomass [12], but very low-diversity tropical forests were not considered in these analyses. The tropical forests in the Hawaiian Islands represent a low-diversity extreme, as a result of its young geological origins [21] and extreme isolation from continental land masses: at approximately 4000 km from the nearest continent, Hawai‘i is the world's most isolated archipelago. The resulting native flora in Hawai‘i is disharmonic (i.e., missing many functional groups) and is about 90% endemic [22]. While long-term plot-based ecological measurements across the tropics have focused on high-diversity forests, there have been surprisingly few data from low-diversity tropical forests [23], [24], [25], [26]. Such low-diversity forests present many interesting contrasts to other tropical forests, and within Hawai‘i they also fall across striking environmental gradients (Table 1).

**Table 1:** Distinctive structural and demographic features of Hawaiian forests.

***Environmental Conditions***
Large variation in elevation, rainfall, temperature and soils among forests that are geographically close ^1–2^
High light levels in intact wet, mesic, and dry forest (1.9–40% diffuse light transmission) ^3–9^
***Species Composition and Diversity Patterns***
A global biodiversity hotspot due to high endemism and number of endangered species ^10–11^
Same species distributed in many habitats differing in environmental conditions, demonstrating exceptional phenotypic plasticity ^10–14^
Tree ferns common and often the understory dominant in wet forests at all elevations, whereas outside of Hawai‘i they tend to be more restricted ^10^
Monodominance by a few canopy species ^15^
***Autecology of Plant Species***
*Metrosideros polymorpha* dominant in wet forests throughout succession (as pioneer and late successional species) ^15–17^
Extremely slow growth of primary pioneer species, *M. polymorpha* (1–2 mm/year diameter) ^18–20^
Nurse logs serve as a substrate for seedling regeneration ^21^
Dieback and regeneration of canopy dominant *M. polymorpha* in cohorts contribute strongly to gap dynamics ^16, 22^
***Trophic Interactions***
Evolution without land mammals ^23, 24^
Documented extinctions of plants, pollinators and dispersers may influence present day evenness and rarity measures ^23^
Animal dispersal of seeds conducted entirely by birds before human contact ^24, 25^
Apparently low rates of insect herbivory ^26^ and seed predation ^27^
Presence of invasive weeds, ungulates, and birds may alter present-day plant-animal interactions ^25–26, 28^

Superscripts refer to references listed in Table S5 in File S2.

The aim of this study was to: 1) characterize and compare two extremely low-diversity Hawaiian forests, montane wet and lowland dry forest, and 2) compare the structural attributes of these two forests to more diverse tropical forests within the Center for Tropical Forest Science (CTFS) permanent plot network. Including the Hawaiian plots as part of a cross-plot analysis allows, for the first time, examination of forest structure along a diversity gradient that varies almost 80-fold across large-scale plots with consistent measurement protocols.

We used the initial census of large-scale permanent plots in Hawai‘i to examine structural and floristic characteristics of two forests that are geographically close but located in widely contrasting environments. The two Hawaiian forest types examined in this first census were montane wet forest (MWF) and lowland dry forest (LDF). Many studies have shown that forests established in areas with higher rainfall or temperature have higher diversity [1], [27], [28], and also greater basal area, tree height, and above-ground biomass [28], [29], [30], [31]. Further, forests in higher rainfall areas tend to have a greater representation of larger trees, but lower tree densities [32]. We therefore ask: 1) How do the two Hawaiian forests compare in terms floristic and life form composition, stand structure, species diversity, and non-native species cover? Our study was not designed to specifically examine the effects of climate on forest structure and composition, but we used this study design, to test a prediction based on the previous literature that Hawaiian dry forest would have greater stem density, lower diversity, and smaller diameter trees than wet forest [32]. To place our findings in a broader context, we also asked: 2) Can the extremely low forests of Hawai‘i have similar structural attributes to more diverse tropical forests? To examine this question, we compared Hawaiian forests with others in the CTFS network enabling the comparison of forest structural variables across a range of environments and diversity levels [1], [28], [31], [33], [34], [35]. If Hawaiian forests converge with other tropical forests, the importance of climate in determining forest structure is highlighted.

## Materials and Methods

### Study Sites

**Figure 1:**
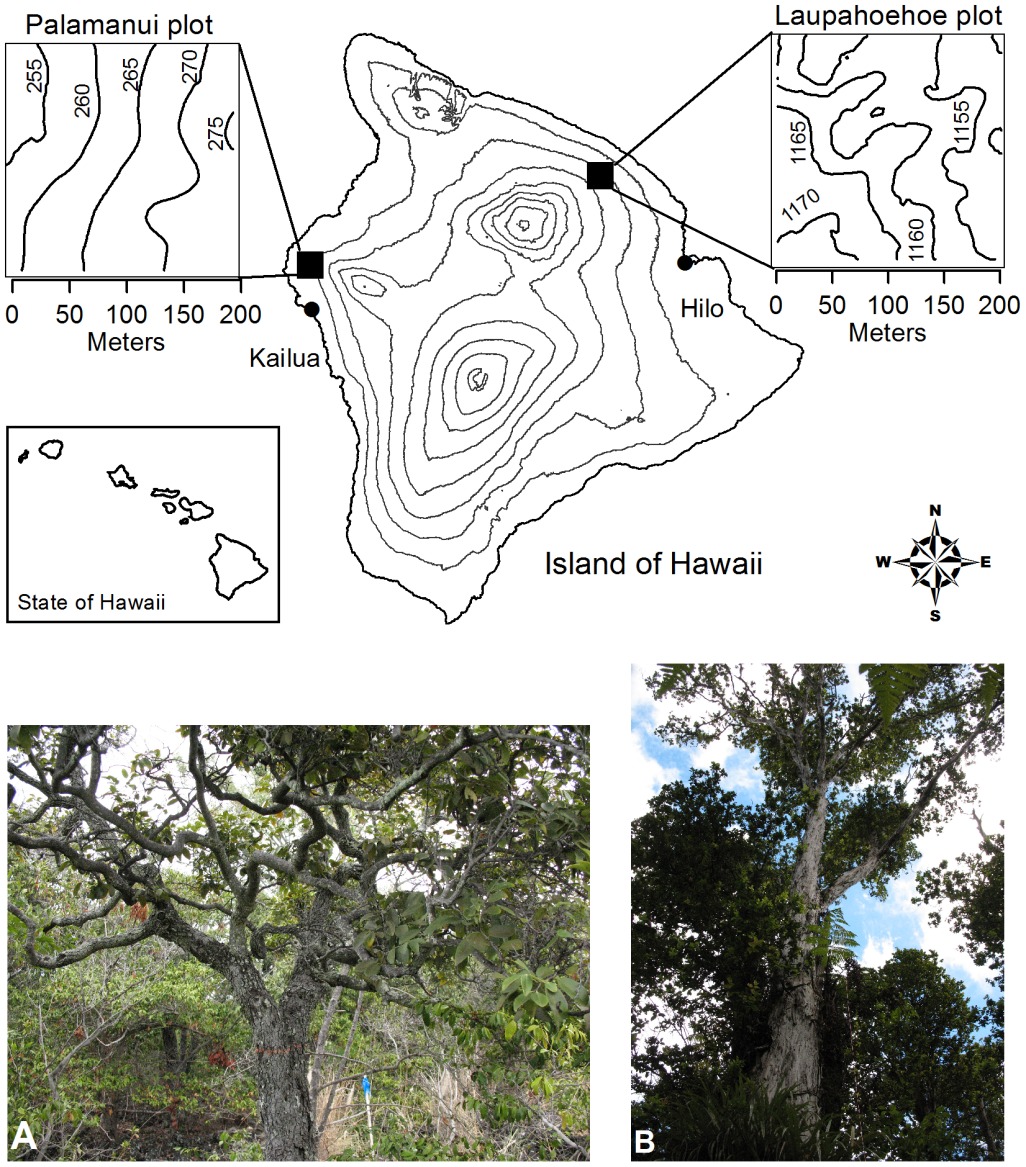
Contour map of the two 4-ha forest plots on Hawai‘i Island. Pālamanui site in west Hawai‘i is lowland dry forest (LDF; left panel showing the dominant canopy tree *Diospyros sandwicensis* and the open canopy and understory structure of small trees and shrubs); Laupāhoehoe plot in east Hawai‘i is montane wet forest (MWF; right panel showing *Metrosideros polymorpha* tree and *Cibotium* spp. tree fern understory).

In 2008 and 2009, we established two forest dynamics plots (FDPs) on Hawai‘i Island – one within montane wet forest (MWF) and one within lowland dry forest (LDF), to initiate the Hawai‘i Permanent Plot Network (HIPPNET; Fig. 1). We focused our study on Hawai‘i Island, because it has the greatest area of intact forests, a complete map of lava flow ages, and excellent infrastructure for ecological studies. As the youngest island in the archipelago (<700,000 years), it has had the least time for plant colonization and subsequent speciation, and thus has lower species richness relative to its size than the older islands [36]. We selected areas in excellent ecological condition that are representative of a given forest type, with high native species cover, and a commitment by ownership to long-term conservation objectives. Notably, all forests in Hawai‘i are affected to some degree by altered trophic interactions due to invasion of non-native species or extinction of the native species [37], but this is not unique to Hawai‘i [38]. Non-native stems that were encountered were measured for percent cover, and then controlled mechanically or chemically (see “*Plot Establishment and Vegetation Measurements*” below) and were not considered in the census of stems.

#### Montane wet forest (MWF)

The 4-ha Laupāhoehoe FDP (19°55' N, 155°17' W) is located within the state-owned Laupāhoehoe Natural Area Reserve section of the Hawai‘i Experimental Tropical Forest (HETF) on the northeast slope of Mauna Kea volcano. Permits were obtained for work in the HETF through the Institute of Pacific Islands Forestry and the Hawai‘i Division of Forestry and Wildlife/Department of Land and Natural Resources. The mean elevation of the plot is 1120 m.a.s.l. with slopes of 0–20%, and the overall direction of downslope is northwards towards the Pacific Ocean. The substrate within the plot is 4000-14,000 years old [39]. Soils were formed from weathered volcanic material, and are deep, rocky, and moderately well-drained silty clay loam in the Akaka series, and classified as hydrous, ferrihydritic, isothermic Acrudoxic Hydrudands (websoilsurvey.nrcs.usda.gov). Rainfall at the MWF is dominated by tradewind-driven precipitation [40]. Interpolated mean annual precipitation, based on analysis of climate station data over 30 years, is 3440 mm with no distinct dry season [41] and mean annual air temperature is 16°C [42]. The forest consists of evergreen broad-leaved trees, and the ∼25–28 m canopy is dominated by *Metrosideros polymorpha* (Myrtaceae; Fig. 1) and to a lesser extent, *Acacia koa* (Fabaceae). Vegetation at the MWF is highly representative of this forest type in Hawai‘i [43] (see references in Table 1).

The dominant pre-human contact disturbance regime in this forest type was single-to multiple-tree falls, with the maximum gap size averaging 21.5 m^2^ [44]. Larger openings coincide with dieback due to cohort senescence of older *M. polymorpha* stands [45]. Following contact, large *A. koa* trees were occasionally harvested for traditional canoe building. In modern times, limited *A. koa* logging occurred in the HETF but was restricted to <100 m of an unimproved road that traverses areas. There is no evidence of logging within the MWF [46], which >500 m from the road. Non-native wild pigs disturb soils while rooting, as well as tree ferns [47], with damage over a large area.

#### Lowland dry forest (LDF)

The 4-ha Pālamanui FDP is an example of one of the world's most endangered forest types, and is located on a privately-owned tract of dry forest on the northwest slope of Hualālai Volcano in the district of North Kona (240 m elevation, 19°44' N, 155°59' W). A memorandum of understanding was established with the land owners and managers, the Palāmani Group, for permission to conduct research in the lowland dry forest site. The mean elevation of the plot is 240 m.a.s.l. Geological substrate in the Pālamanui area consists of ‘a‘ā lava with scattered pāhoehoe flows dating to 1,500–3,000 years old [48]. Soils developing at this site are shallow, rocky, highly organic, and classified as euic, isothermic, shallow Lithic Ustifolist (websoilsurvey.nrcs.usda.gov). Interpolated mean annual precipitation at the LDF site is 835 mm [41], [49], with large within- and between-year variability [50]. For the LDF, major rainfall events typically occur in the winter as low pressure storms (“Kona lows”) while summers tend to be dry and characterized by small convective storms. Mean daily air temperature is approximately 20°C (wrcc.dri.edu). Native vegetation consists of evergreen broad-leaved trees and shrubs that form an open-canopy forest that reaches heights of ∼7–8 m dominated by *Diospyros sandwicensis* (Ebenaceae) and *Psydrax odorata* (Rubiaceae; Fig. 1). One species (*Erythrina sandwicensis*) is drought deciduous and is only represented by a few individuals.

Pre-contact disturbance regimes likely included tree falls. Following contact, selective harvesting of valuable woods (e.g., sandalwood) occurred throughout the area but we do not know of any logging that occurred within the plot. In the last 200 years, much of the lowland dry forest in Hawai‘i has been subjected to grazing and browsing by exotic ungulates, with remnants impacted by wildfire carried by non-native grasses [51]. These factors have reduced the native forest to a fraction of its original extent [52]. While the area containing the FDP has not been burned or significantly browsed by ungulates, the surrounding area is a matrix of degraded LDF and open grassland, and in 2009, a fence and firebreak were installed around the area to protect it from ungulates and fire.

### Plot Establishment and Vegetation Measurements

We applied field methodology developed by the Center for Tropical Forest Science global FDP network [53]. Both of our 4-ha FDPs (200×200 m) were oriented north-south and located at the center of a 16 ha buffer area, with all edges at least 100 m from any road or major trail where possible. From 2008 to 2009, we tagged all live, native woody plants ≥1 cm diameter at breast height (DBH, at 130 cm), and mapped tagged plants relative to 5 m×5 m grids installed throughout the plots. Each tagged plant was identified to species and measured for DBH. More detailed methods are in Methods S1 in File S1.

Finally, we estimated and mapped cover of abundant non-native herbaceous, shrub and tree species, which will be important for understanding long-term vegetation change. At each site, we chose six abundant focal species or life forms that were considered “invasive pests” according to their Hawai‘i Weed Risk Assessment scores (Daehler 2004; www.botany.hawaii.edu/faculty/daehler/wra/full_table.asp). Percent cover within each 5×5 m subquadrat was estimated in the following categories: 0: absent, 1: <5%, 2: 5–25%, 3: 25–50%, 4: 50–100%. Non-native trees with stems ≥1 cm at 130 cm were individually mapped. The DBH of the largest stem of non-native trees <5 cm was estimated to the nearest centimeter and measured to the nearest centimeter if >5 cm. For trees with multiple stems, we counted the total number of stems ≥1 cm at 130 cm. After the non-native trees were mapped, they were girdled and sprayed with herbicide. We did not spray herbicide on the grasses in the LDF, nor the vine *Passiflora tarminiana* in the MWF.

### Data Analyses

#### Stand structure

We determined stand structural characteristics based on DBH measurements. We considered multiple-stemmed plants as single individuals for the calculation of stem density, and summed the basal area of all stems for the calculation of basal area (m^2^/ha). For each species, we calculated relative abundance (RA, %) as the number of individuals of that species/total number of individuals, relative dominance (RD, %) as the basal area of that species/total basal area, and relative frequency (RF, %) as the number of quadrats with that species/total number of quadrats.

#### Above-ground biomass

To estimate above-ground biomass (AGB) for the two plots, we used site-specific and species-specific information whenever possible for wood specific gravity, tree height, and DBH (equations derived from 52,54,55,56; see Table S1 in File S2). When these were not available, we compiled data from global databases, utilizing equations based on other sites, and in some cases for other species from within the same genera [56], [57]. Previous studies have reported that genus means are reasonable proxies for species values for specific gravity (*r* ^2^>0.70; [58], [59]).

To determine tree height, we applied species-specific equations of [54] giving the relationship of tree height vs. DBH, to each individual tree for 12 of the MWF species and 4 of the LDF species (Table S1 in File S2). For the other species, we used the general wet and dry forest equations [55] to determine tree height. We used these tree height estimates to calculate AGB for each tree using published equations that also included DBH and wood specific gravity. Hawai‘i-specific equations for AGB were available for 5 MWF species and 4 LDF species. For another two species of the LDF, *D. sandwicensis* and *P. odorata*, equations were available that were developed specifically from our study site [52] (Table S1 in File S2).

#### Species richness and diversity

Species area curves were generated by plotting cumulative number of species against area for the 20 m×20 m quadrats. Rarefaction analyses were based on 999 permutations (PRIMER-E v. 6, PRIMER-E Ltd, Plymouth, UK), which randomized the sampling order and resulted in a robust average curve. We present several indices: Sobs (the observed number of species), Chao 1 based on rare species (non-parametric), and Michaelis-Menten (parametric), given uncertainty in the ideal estimator [60], [61], [62]. We used the program EstimateS 9.1.0 to calculate species diversity indices and an estimate of error. We report Fisher's alpha, Shannon diversity index, and Simpson's index (inverse form) following standard formulas [63]. Overlap in species composition between the two sites was determined using the Sørenson similarity index (SI): 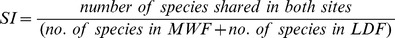



#### Forest type comparisons

We compiled data for 19 additional mainland and island CTFS tropical plots for which climate and structure data were available (Table 2). Differences between the Hawai‘i plots and other CTFS plots were assessed using one sample *t*-tests. Differences in the characteristics of island and mainland plots were assessed using Wilcoxon signed-rank tests [64]. These statistics were analyzed with JMP v. 6 [29], [65].

**Table 2:** Diversity and forest structure characteristics of plots in the Center of Tropical Forest Science global plot network, including the Hawaiian plots, arranged in order of descending species richness.

CTFS plot location	Plot code	Latitude	Mean annual rain (mm)	Dry season months	Mean elevation (m)	Mean annual temp (°C)	Land type	Plot size (ha)	No. of species	No. of families	Mean species/family	Fisher's α/ha	H'/ha	Trees/ha	Basal area (m^2^/ha)	Dom. of most common family	Above-ground biomass (Mg/ha)^7^
Lambir, Malaysia	LAM	4.19	2664	0	170	26.6	I	52	1182	83	14.2	165	2.40	6915	43.5	41.0	497.2
Yasuní, Ecuador	YAS	−0.69	3081	0	230	28.4	M	50	1114	81	13.8	187	2.44	3026	33.0	14.9	282.4
Pasoh, Malaysia	PAS	2.98	1788	1	80	28.0	M	50	814	82	9.93	124	2.31	6708	31.0	28.2	339.8
Khao Chong, Thailand	KHA	7.54	19851	32	140	27.3	M	24	593	na	na	na	Na	5063	na	na	na
Korup, Cameroon	KOR	5.07	5272	3	200	26.7	M	50	494	62	7.97	48.0	1.75	6580	32.0	16.1	na
Ituri, Dem. Rep. of Congo3	ITU	1.44	1700	3–4	780	23.1	M	404	445	na	na	na	Na	7200	na	na	na
Palanan, Philippines	PAL	17.04	3379	4–5	110	23.5	I	16	335	60	5.58	43.4	1.92	4125	39.8	52.8	290.1
Bukit Timan, Singapore	BUK	1.25	2473	0	150	26.9	I	2	329	62	5.31	60.0	1.90	5950	34.5	38.4	na
BCI, Panama	BCI	9.15	2551	3	140	26.9	M	50	299	58	5.16	34.6	1.62	4168	32.1	11.4	306.5
Mo Singto, Thailand	MOS	14.43	2200	5	770	23.0	M	30.5	262	na	na	na	Na	Na	na	na	na
Huai Kha Khaeng, Thailand	HKK	15.63	1474	6	590	24.1	M	50	251	58	4.33	23.3	1.50	1450	31.2	21.2	211.2
La Planada, Colombia	LPL	1.16	4084	0	1840	19.0	M	25	240	54	4.44	30.6	1.72	4216	29.8	14.9	177.6
Dinghushan, China	DIN	23.16	1985	0	350	20.9	M	20	210	na	na	na	Na	3581	na	na	na
Sinharaja, Sri Lanka	SIN	6.4	5016	0	500	22.6	I	25	204	46	4.43	24.4	1.53	7736	45.6	26.7	357.9
Doi Inthanon, Thailand	DOI	18.52	1908	6	1700	21.4	M	15	192	na	na	na	Na	4913	na	na	na
Luquillo, Puerto Rico	LUQ	18.33	3548	0	380	22.8	I	16	138	47	2.94	13.5	1.45	4194	38.3	17.3	276.1
Nanjenshan, Taiwan	NAN	22.06	3582	0	320	23.5	I	3	125	41	3.05	15.6	1.64	12133	36.3	32.3	na
Ilha do Cardoso, Brazil5	ILH	−25.1	2100	0	5	22.4	M	10	106	na	na	na	Na	Na	na	na	na
Mudumalai, India	MUD	11.6	1250	6	1050	22.8	M	50	72	29	2.48	6.20	0.944	510	25.5	28.2	174.2
Laupāhoehoe, USA	LAU	19.93	3440	1	1150	16.0	I	4	21	15	1.40	2.58	1.82	3078	67.36	37.4	247.9
Pālamanui, USA	PLN	19.74	835	12	240	20.0	I	4	15	15	1.00	1.18	1.00	3487	8.6	74.2	29.4

Southern hemisphere latitudes are negative; land types are island (I) and mainland (M); dry season months are as those with <100 mm precipitation (Richards 1996). Data are from [104] and ctfs.si.edu unless indicated by footnotes.

1 Mean annual rainfall data for the nearby city of Songkhla, Thailand (www.world-climates.com)

2 Kira T (1998) NPP Tropical Forest: Khao Chong, Thailand, 1962–1965. Data set. Available on-line [http://www.daac.ornl.gov] from Oak Ridge National Laboratory Distributed Active Archive Center, Oak Ridge, Tennessee, U.S.A.

3 Average of 4 plots (2 monodominant forest, 2 mixed forest)

4 Divided into four 10-ha plots

5 Data from Ferreira de Lima RA, Oliveira AAD, Martini AMZ, Sampaio D, Souza VC, Rodrigues RR (2011) Structure, diversity, and spatial patterns in a permanent plot of a high restinga forest in Southeastern Brazil. Acta Botanica Brasilica 25: 633–645.

6 Basal area including tree ferns; 36.1m^2^/ha without tree ferns

7 Chave J and 37 others (2008) Assessing evidence for a pervasive alteration in tropical tree communities. PLoS Biology 6: 455–462.

## Results

### Comparison of Floristics and Life Forms in Hawaiian Forests

The two Hawai‘i forests were distinct in floristic composition (Table S2 in File S2). The plots had a very low Sørenson similarity index of 0.06 (a value of 1 would indicate complete overlap). Only *M. polymorpha* occurred in both forests; it was the second most common species in MWF but was represented by only 5 individuals in the 4-ha LDF plot. Species richness was 21 in the MWF and 15 in the LDF. Fifteen families were represented at each site, and the canopy trees at the two sites were from different families, though four families were represented in the understory or the midstory at both sites (Euphorbiaceae, Fabaceae, Myrtaceae, and Rubiaceae).

**Figure 2:**
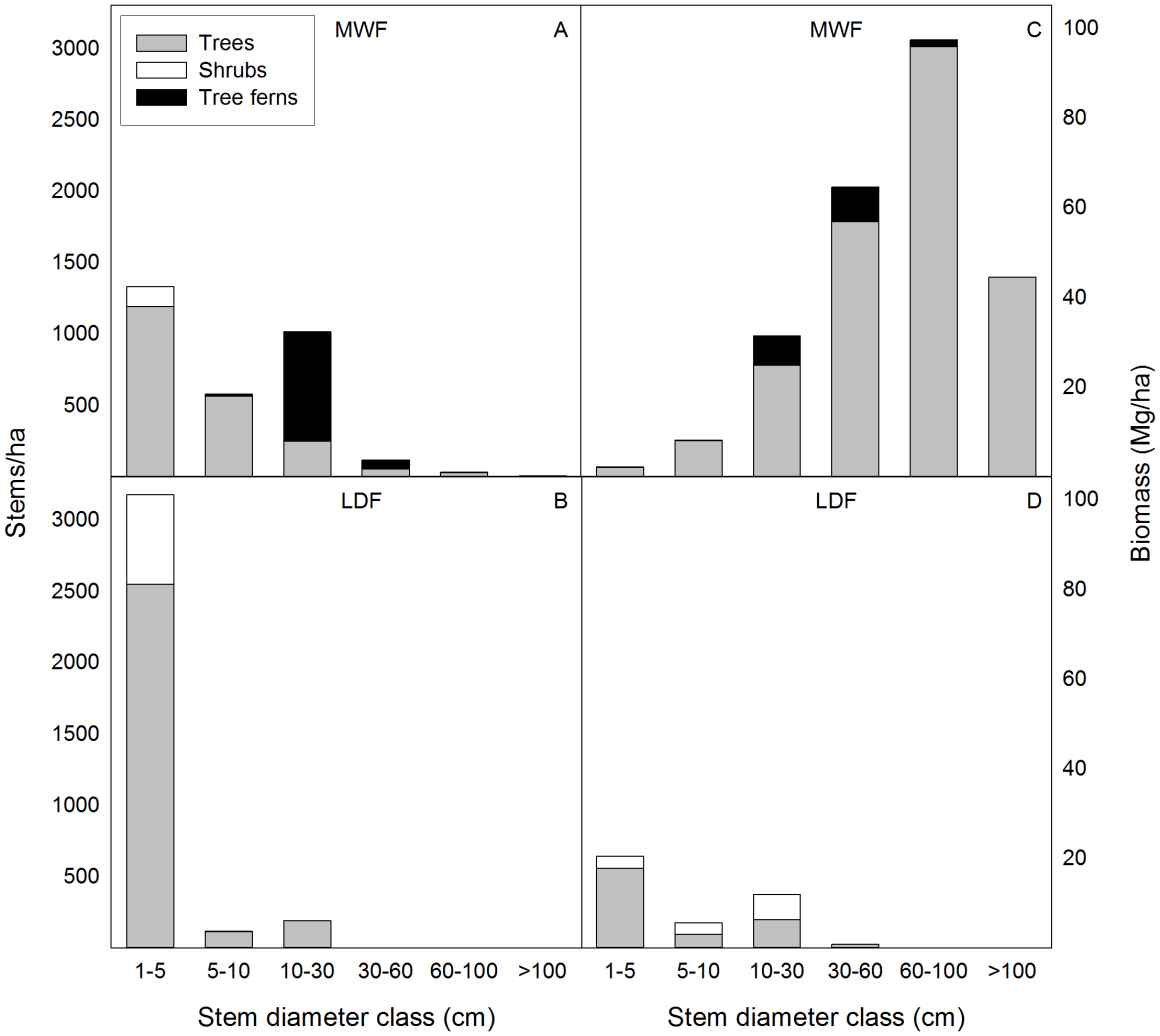
Life form distribution of stems and biomass by diameter size intervals. In (A) Hawaiian montane wet forest (MWF) and (B) lowland dry forest (LDF), stems represent the number of main stems (i.e., one per individual, not including other multiple stems). In (C) MWF and (D) (LDF), biomass calculations were made for all stems (including multiple stemmed individuals). Diameter classes are 1–4.99 cm, 5 - <9.99 cm, 10–29.99 cm, 30–59.99 cm, 60–99.99 cm, and ≥100 cm.

The plots differed in their distribution of plant life forms. In the MWF, 68% of stems were trees, 4.5% were shrubs, and 28% were tree ferns, accounting for 45%, 8.3% and 46% of the basal area respectively. In the LDF, 82% of stems were trees and 18% were shrubs, accounting for 95% and 5% of basal area respectively (Fig. 2). In the MWF, a large proportion of stems (31%) were growing on non-soil substrates, primarily tree ferns, logs or rocks, with 17% of all individuals growing on dead tree ferns (Table S3 in File S2). In contrast, in the LDF, all trees were growing on soil or broken lava, and tree ferns were absent.

### Comparison of Stand Structure in Hawaiian Forests

The MWF had larger trees and lower stem density than the LDF (3078±1.21 and 3487±1.40 stems/ha respectively; Table 3). The tree size class distributions differed between the two forests as expected based on their contrasting climates: the LDF had mainly small stems and the MWF had a much more even spread of size classes (Fig. 2). Because the stems in the LDF were small, total basal area and biomass values were low. Thus, the MWF had a nearly eight-fold higher basal area than the LDF (67.3 vs. 8.6 m^2^/ha respectively; Tables 3–4), and tree ferns accounted for 31.2 m^2^/ha basal area. Above-ground biomass in the MWF was also more than eight times higher than the LDF (248 Mg/ha vs. 29.4 Mg/ha respectively; with 15.6 Mg/ha in the MWF accounted for by tree ferns; Table 5). The above-ground biomass value for the MWF was consistent with that previously estimated for surrounding forest in the same reserve [66]. In both forest types, the two most common canopy species represented 87–88% of biomass (Table 5). In the MWF, the very large trees (≥60 cm) made up the greatest proportion of the biomass, but in the LDF the majority of the biomass was in the 1–5 cm size class (Fig. 2). More multi-stemmed individuals make up the LDF, a mean of 3.2 stems/individual, compared to 1.4 stems/individual in the MWF plot) (Table S4 in File S2).

**Table 3:** Statistics on abundance, basal area, and frequency of the species in the Laupāhoehoe (montane wet forest) plot, with data displayed on an absolute and a relative basis.

Laupāhoehoe montane wet forest							
Species	No. individuals	Basal area (m^2^/ha)	Presence (no. of quadrats)	Relative abundance (%)	Relative dominance (%)	Relative frequency (%)	IV (%)
METPOL	2631	25.2	100	21.4	37.5	10.2	69.1
CIBGLA	2274	17.7	100	18.5	26.4	10.2	55.1
CHETRI	3320	4.17	100	27.0	6.20	10.2	43.4
CIBMEN	1076	13.2	100	8.74	19.6	10.2	38.6
COPRHY	972	0.585	99	7.90	0.870	10.1	18.9
ILEANO	965	0.466	99	7.84	0.692	10.1	18.7
ACAKOA	141	5.49	57	1.15	8.16	5.84	15.1
BROARG	271	0.0454	74	2.20	0.067	7.58	9.85
MYRLES	237	0.0571	70	1.93	0.085	7.17	9.18
VACCAL	255	0.0328	51	2.07	0.0488	5.23	7.35
HEDHIL	43	0.0200	29	0.349	0.0297	2.97	3.35
PERSAN	35	0.0084	28	0.284	0.0125	2.87	3.17
CIBCHA	34	0.232	17	0.276	0.345	1.74	2.36
CLEPAR	19	0.00230	17	0.154	0.00342	1.74	1.90
MELCLU	13	0.00235	11	0.106	0.00349	1.13	1.24
PSYHAW	10	0.00219	9	0.0812	0.00326	0.922	1.01
MYRSAN	6	0.00166	6	0.0487	0.00246	0.615	0.666
PIPALB	4	0.00226	4	0.0325	0.00335	0.410	0.446
TREGRA	2	0.000107	2	0.0162	0.000159	0.205	0.221
LEPTAM	2	0.0000682	2	0.0162	0.000101	0.205	0.221
ANTPLA	1	0.000154	1	0.0081	0.000229	0.102	0.111
***Total***	***12311***	***67.3***					

**Table 4:** Statistics on abundance, basal area, and frequency of the species in the Pālamanui (lowland dry forest) plot, with data displayed on an absolute and a relative basis.

Pālamanui lowland dry forest							
Species	No. individuals	Basal area (m^2^/ha)	Presence (no. of quadrats)	Relative abundance (%)	Relative dominance (%)	Relative frequency (%)	IV (%)
DIOSAN	2208	6.41	99	15.8	74.2	18.3	108.3
PSYODO	8640	1.27	100	62.0	14.7	18.5	95.2
DODVIS	2301	0.359	94	16.5	4.15	17.4	38.1
SOPCHR	5	0.21	4	0.0359	2.38	0.741	3.20
SANPAN	275	0.156	32	1.97	1.81	5.93	9.70
OSTANT	147	0.0900	40	1.05	1.04	7.41	9.50
WIKSAN	88	0.0890	44	0.631	1.03	8.15	9.81
EUPMUL	134	0.023800	54	0.961	0.275	10.0	11.2
SENGAU	70	0.013300	27	0.502	0.154	5.00	5.66
METPOL	12	0.013	9	0.0860	0.152	1.67	1.90
MYOSAN	54	0.007480	26	0.387	0.087	4.81	5.29
SIDFAL	1	0.0008	1	0.0072	0.00947	0.185	0.202
PLEHAW	1	0.0007	1	0.0072	0.00843	0.185	0.201
ERYSAN	2	0.000513	2	0.0143	0.00594	0.370	0.391
PITTER	8	0.00048	7	0.0574	0.00551	1.30	1.36
***Total***	***13946***	***8.64***					

Presence based on 100 20×20 m quadrats per plot; species sorted by importance value (IV), which is the sum of the three relative measures (max 300%); species abbreviations as in Table S2 in File S2.

**Table 5:** Aboveground biomass listed by species for the two Hawai‘i forest plots; species abbreviations as in Table S2 in File S2.

Laupāhoehoe montane wet forest			Pālamanui lowland dry forest		
Species	Biomass (Mg/ha)	Relative biomass (%)	Species	Biomass (Mg/ha)	Relative biomass (%)
METPOL	186	74.9	PSYODO	15.3	51.9
ACAKOA	31.1	12.5	DIOSAN	10.5	35.8
CHETRI	12.4	4.99	METPOL	1.40	4.78
CIBMEN	10.9	4.39	DODVIS	0.921	3.14
CIBGLA	4.55	1.83	OSTANT	0.525	1.79
COPRHY	1.59	0.64	SANPAN	0.359	1.22
ILEANO	1.27	0.51	WIKSAN	0.181	0.615
CIBCHA	0.184	0.07	MYOSAN	0.109	0.372
MYRLES	0.109	0.04	SOPCHR	0.0446	0.152
VACCAL	0.0947	0.04	SENGAU	0.0398	0.135
HEDHIL	0.0456	0.02	EUPMUL	0.0199	0.068
BROARG	0.0394	0.02	PITTER	0.00435	0.0148
PERSAN	0.0124	0.00499	PLEHAW	0.00217	0.00740
PSYHAW	0.00384	0.00155	SIDFAL	0.00187	0.00637
MELCLU	0.00343	0.00138	ERYSAN	0.000871	0.00297
CLEPAR	0.00327	0.00132			
MYRSAN	0.00319	0.00128			
PIPALB	0.00274	0.00110	***Total***	***29.4***	
ANTPLA	0.000287	0.000116			
LEPTAM	0.00017	0.0000685			
TREGRA	0.0000703	0.0000283			
***Total***	***247.9***				

### Comparison of Community Structure in Hawaiian Forests

**Figure 3:**
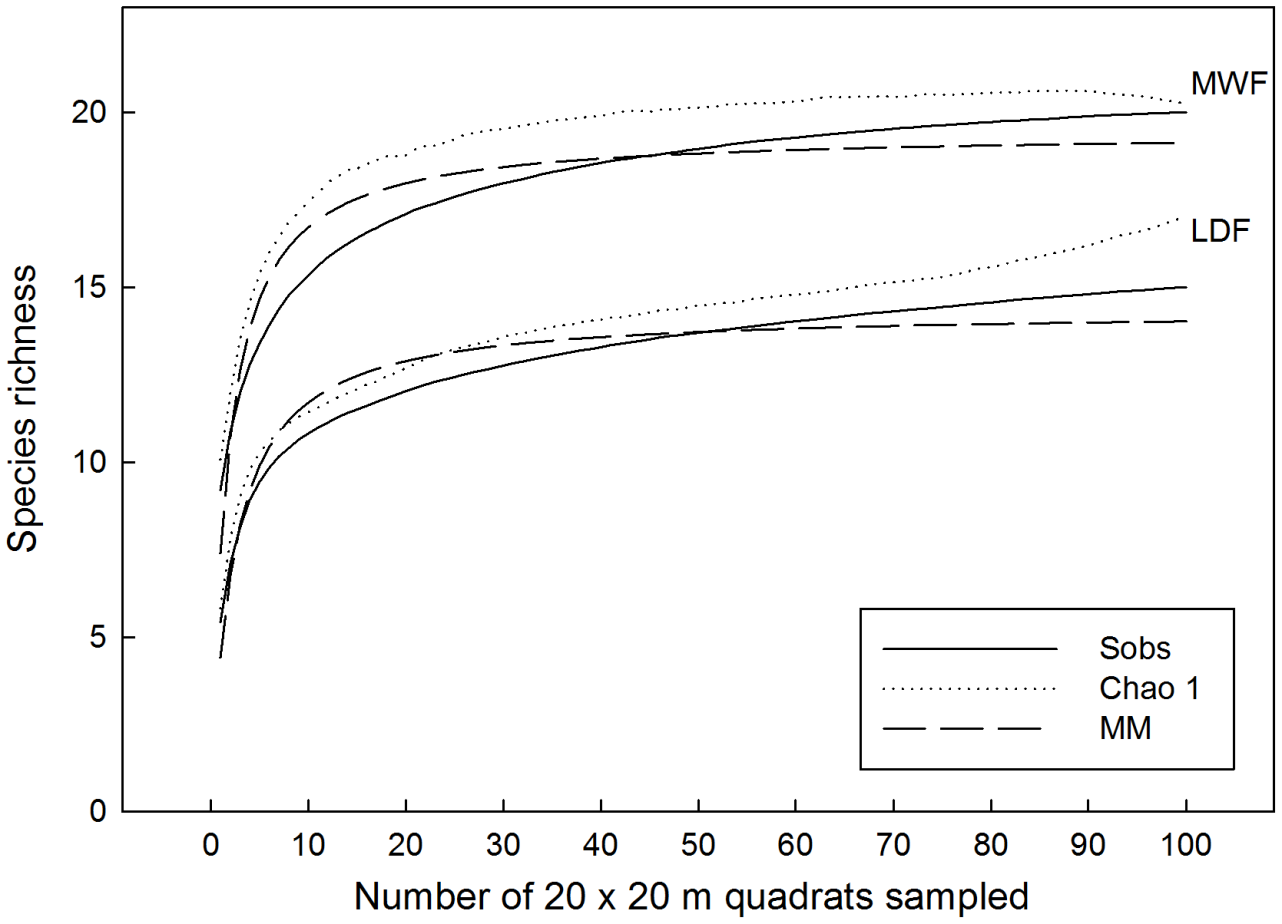
Species accumulation curves. Species number is shown cumulatively, as additional 20 m×20 m quadrats are sampled, until the entire 4-ha plot is represented (100 quadrats), for Hawaiian montane wet forest (MWF) and lowland dry forest (LDF). Three rarefaction techniques are used: Sobs (observed species number), Chao 1, and MM (Michaelis-Menten).

**Figure 4:**
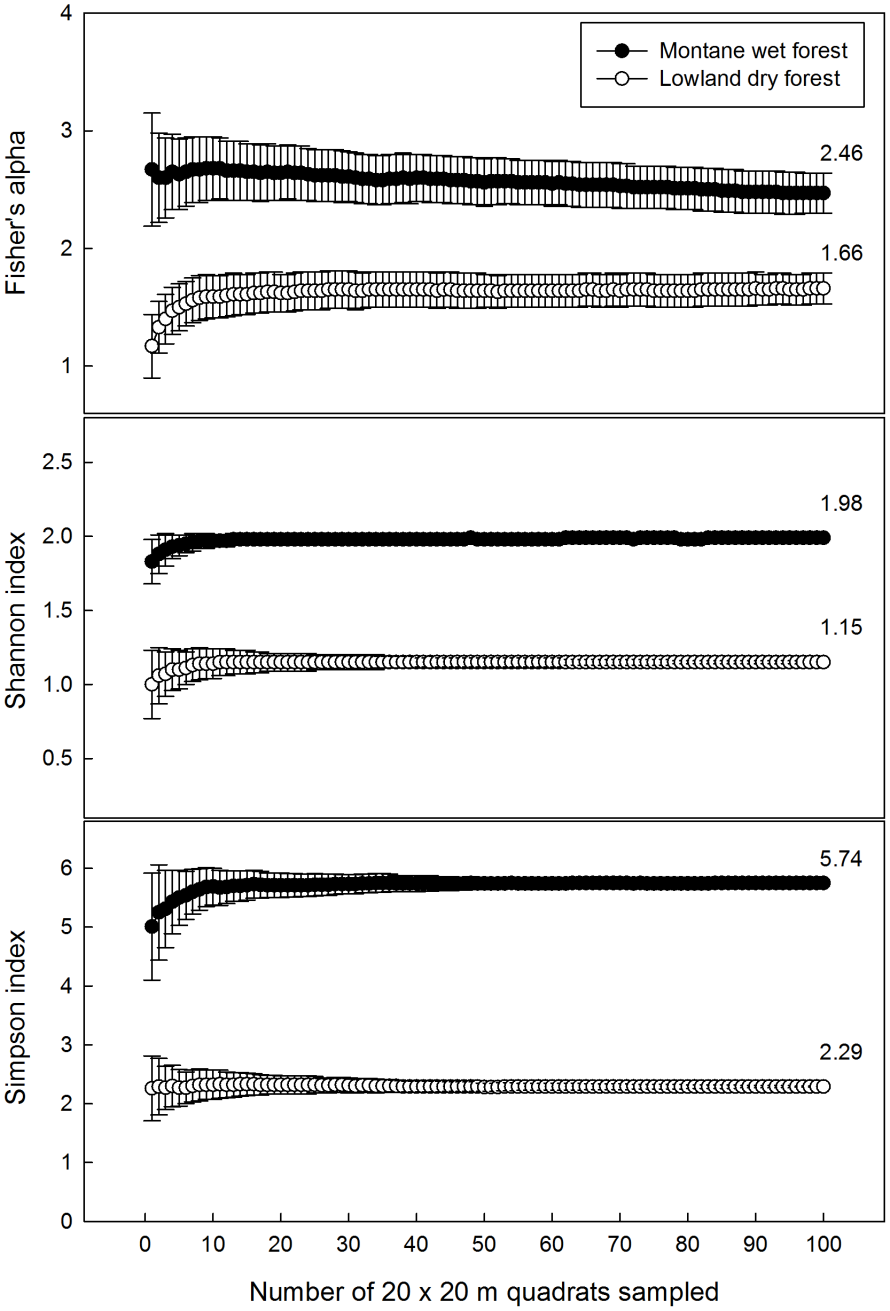
Species diversity indices. Fisher's alpha, Shannon index, and Simpson index for the Hawaiian montane wet forest (MWF) and lowland dry forest (LDF). Each 20×20 m subplot is shown, with the values being cumulative and number above each line representing the entire plot area (4-ha). Values are the diversity index and standard deviation, as estimated by the program EstimateS.

For both the Hawaiian MWF and LDF, rarefaction curves indicated that a 1 ha sample was sufficient to capture 90% of the species present in the larger 4-ha area (Fig. 3). In the MWF, diversity values for the plot were 2.46 (Fisher's alpha), 1.98 (Shannon), and 5.74 (Simpson); in the LDF values were 1.66 (Fisher's alpha), 1.15 (Shannon), and 2.29 (Simpson). When viewed graphically, there was no overlap in any index value between the two forests (Fig. 4): the Hawaiian MWF was more diverse than the LDF. In the MWF, species evenness was higher than in the LDF, primarily because *P. odorata* in the LDF had a relative abundance of over 60%. In contrast the forests were similar in the abundance of uncommon species, and ∼20% of species were rare, i.e., having ≤1 stem/ha (Tables 3–4).

### Non-native Species in the Plots

**Figure 5:**
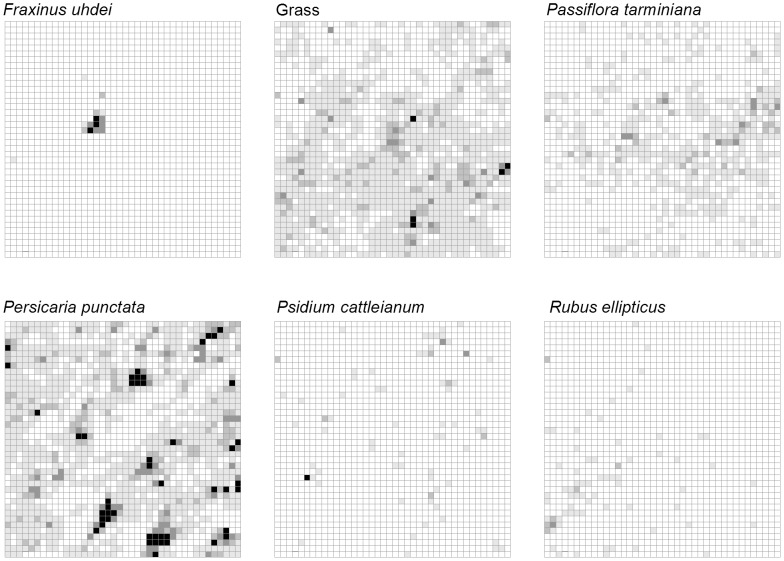
Invasive species cover distribution. Map showing percent cover and locations of invasive species in the MWF. Each grid square represents one 5×5-m subquadrat white: absent, light grey: present to <5%, medium grey: 5–25%, dark grey: 25–50%, black: >50% cover).

**Figure 6:**
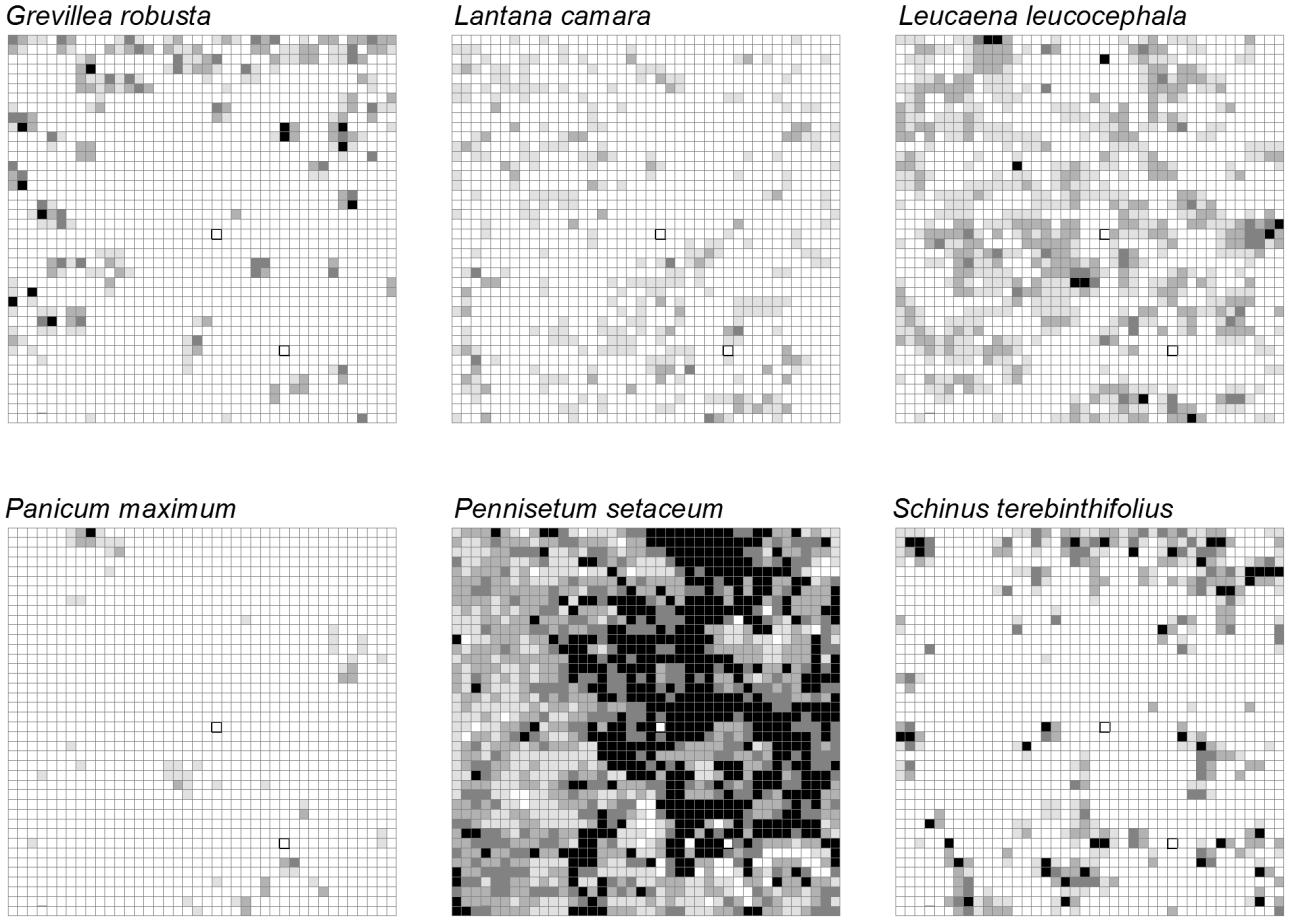
Map showing percent cover and locations of invasive species in the LDF. Each grid square represents one 5×5-m subquadrat (white: absent, light grey: present to <5%, medium grey: 5–25%, dark grey: 25–50%, black: >50% cover).

**Figure 7:**
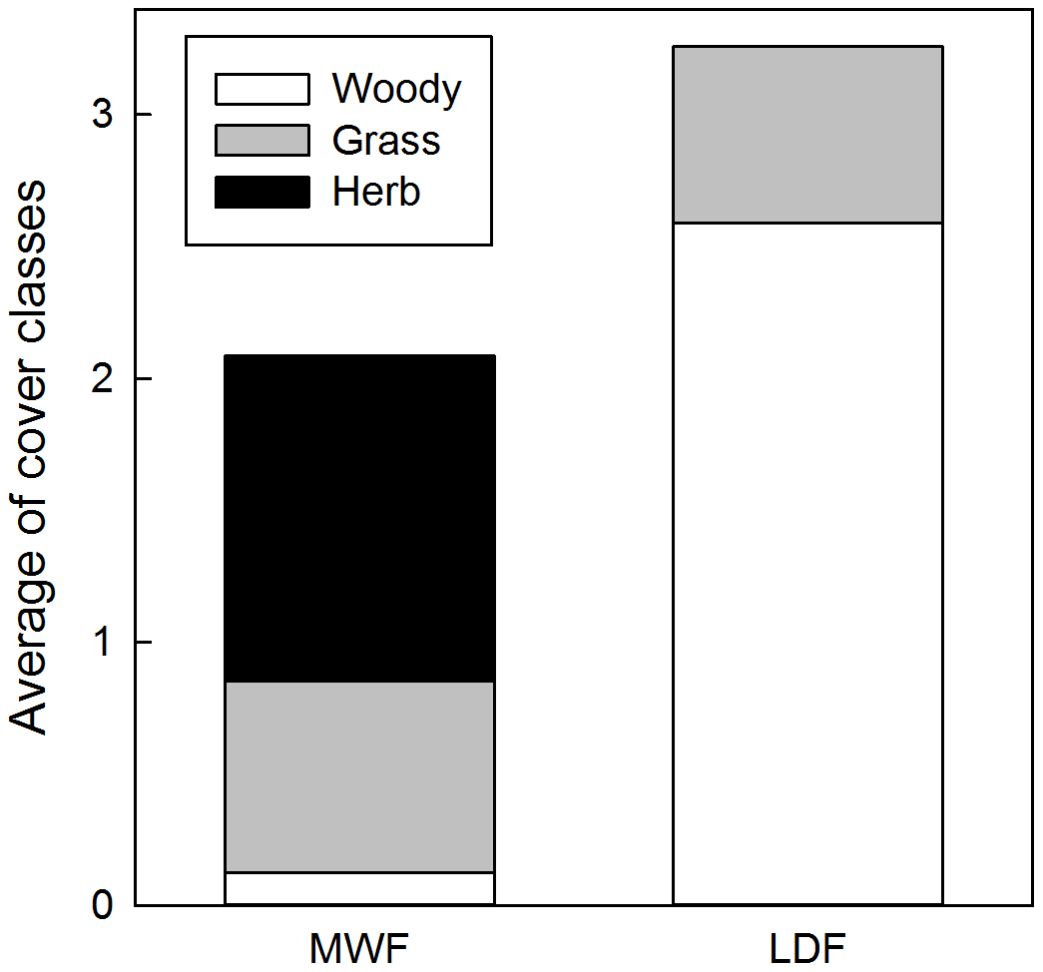
Combined invasive species cover. In each 5×5 m subquadrat a cover score from 0–4 was given based on cover classes (see Methods). The y axis represents the average cover class across the 400 subquadrats, separated by life form: grasses, herbaceous, or woody (shrubs and trees). The combined cover represents the species shown in Figure 5.

Invasive species made up a larger presence in LDF than MWF (Figs.5–6). The grass *Pennisetum setaceum* was most widespread in the LDF and the herbaceous weed *Persicaria punctata* was most common in the MWF, where it tended to dominate low-lying boggy areas. In the LDF there was a greater overall weed cover, particularly of woody weeds (Fig. 7; see also Methods S1 in File S1). In the MWF there were only a few stems that qualified for DBH measurements (>5 cm). *F. uhdei* averaged 32.3 cm (n = 1) and *P. cattleianum* averaged 6.2 cm (n = 1). In the LDF, average DBH for *Grevillia robusta* was 20.0±8.2 cm SE (n = 27), for *Leucaena leucocephalum* it was 5.9±0.8 cm SE (n = 7), and for *Schinus terebinthius* it was 11.0±1.5 cm SE (n = 20).

### Comparison of Hawai‘i to Other CTFS Plots

**Figure 8:**
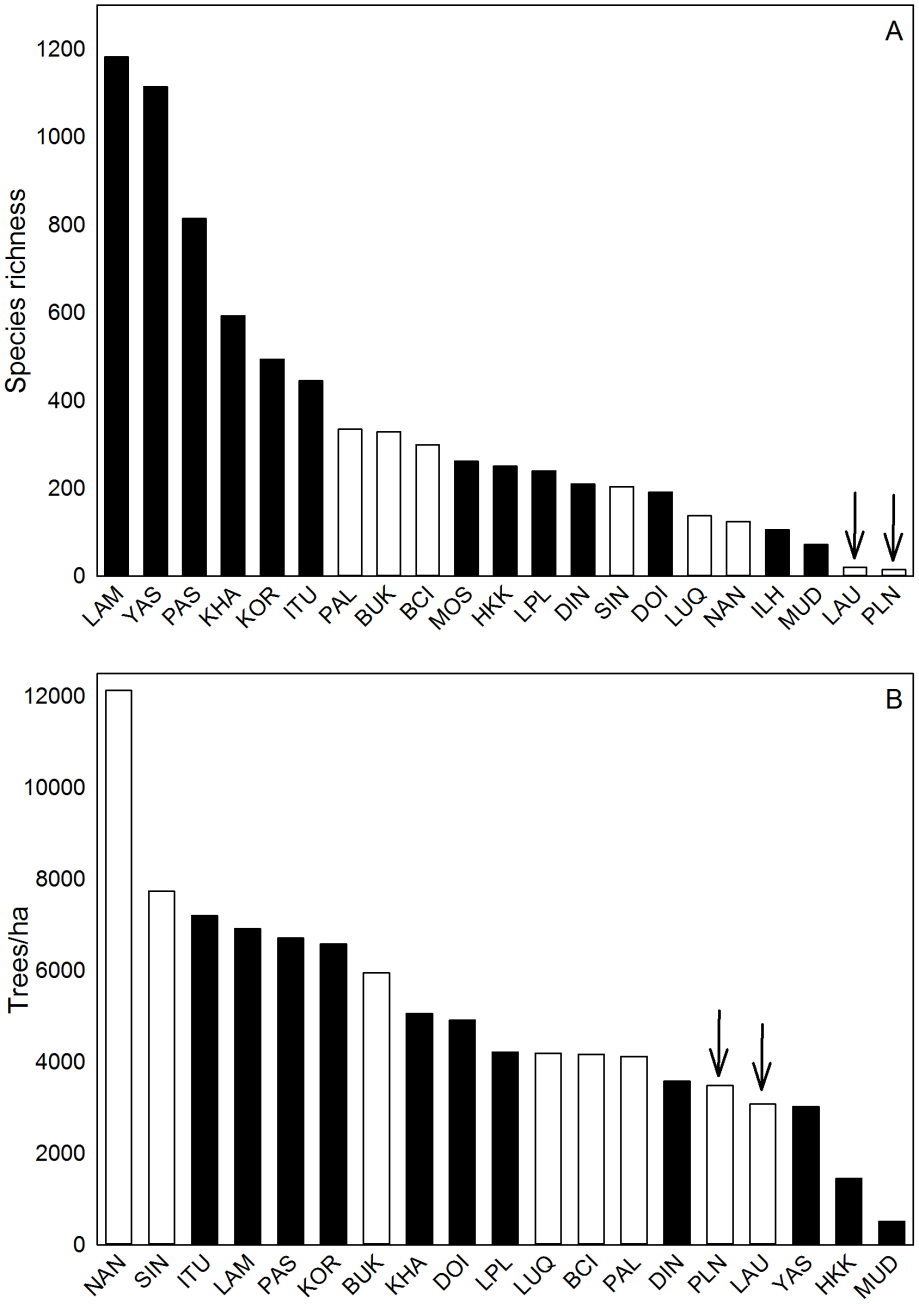
Comparisons of species richness and stem density across a series of CTFS plots. Black bars represent continents and open bars represent islands. Abbreviations as in Table 2. Data from Losos and Leigh, Jr. (2004) and www.ctfs.si.edu.

Diversity of both Hawai‘i forests was very low relative to other CTFS forests, including those on islands and with dry climates (Fig. 8). Across the CTFS network, the mean Fisher's alpha per ha ± SE was 59.7±16.6 (n = 13), and the two Hawaiian forests were statistical outliers, with diversity values more than 2 SD lower (*t* = 3.45 and 3.53, *P*<0.005). The MWF had approximately 15% as many species as the most comparable island site with tropical wet forest (Luquillo, Puerto Rico). Compared with the next two driest CTFS sites, the Hawai‘i LDF had 21% of the number of species found in the Mudumalai, India plot and just 6% of the number of species found at the Huai Kha Khaeng, Thailand plot (Table 2).

**Figure 9:**
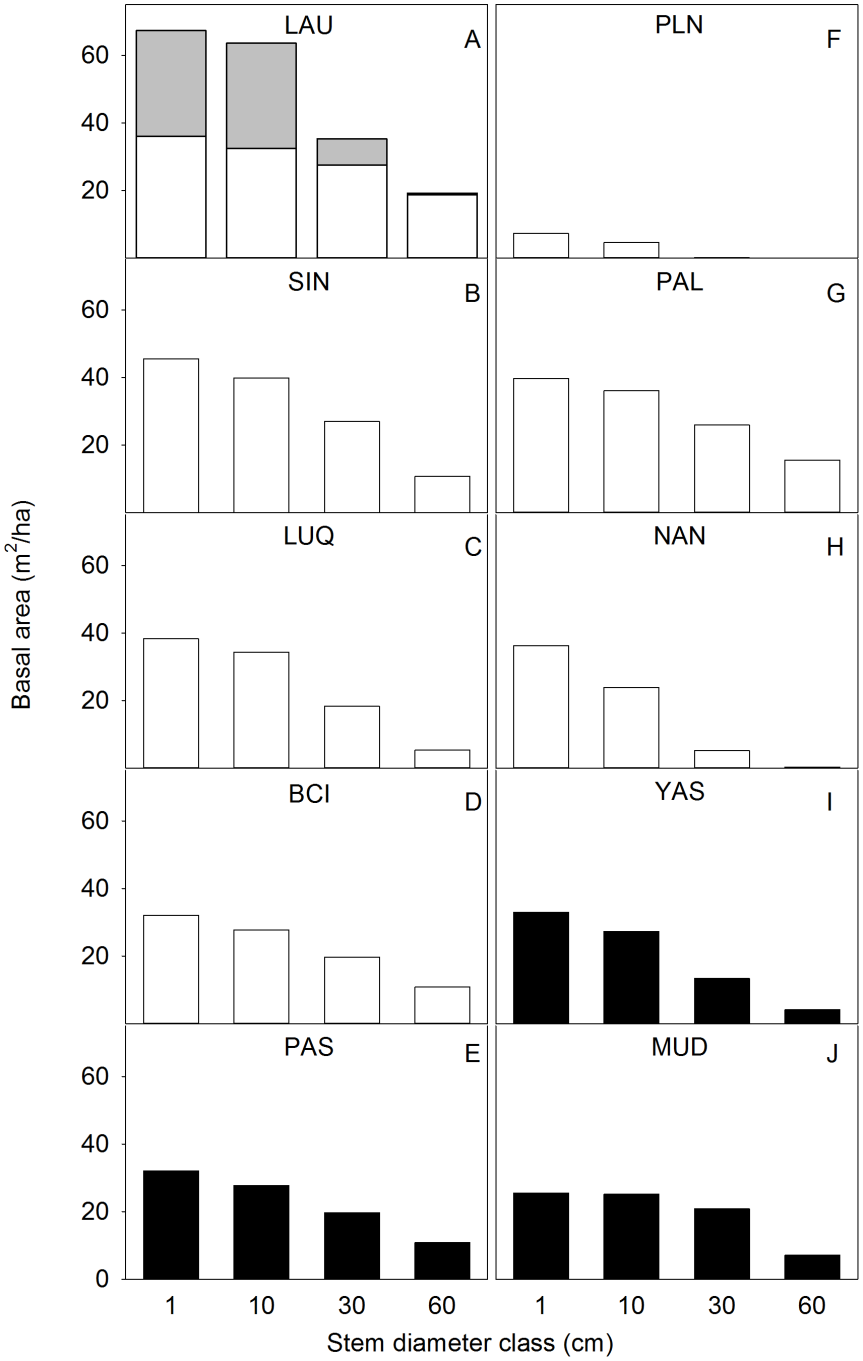
A reverse-cumulative distribution of basal area by size class. Size classes are: ≥1 cm, ≥10 cm, ≥30 cm, and ≥60 cm. Data shown for the Hawaiian montane wet forest (LAU) and lowland dry forest (PLN) (top row) and for selected other CTFS plots. Tree ferns (found only at LAU) are symbolized by the gray bars. Island sites are open bars and continental sites are filled bars. Abbreviations as Table 2. Data from Losos and Leigh, Jr. (2004) and www.ctfs.si.edu.

In contrast to biodiversity, the structural comparisons across the CTFS network revealed complicated patterns. The MWF was similar to other CTFS sites with respect to tree size class distribution (Fig. 9), and was not significantly different from other CTFS plots with respect to standing above-ground biomass/ha. However, the MWF had 35% lower stem density than the all-forest mean of 4733±722 SE (*t* = 2.29, *P* = 0.039, n = 14). Further, the MWF had a 92% higher basal area than the mean of other tropical FDPs due to its high tree fern abundance (*t* = −21.6, *P*<0.0001). When tree ferns were excluded, the basal area of the Hawaiian MWF was within the range of that for other FDPs. For the LDF, stem density was not significantly different than the all-forest mean (*t* = 1.79, *P* = 0.097, n = 16, Table 2), but the LDF was an outlier with its very low basal area (*t* = 10.40, *P*<0.001, n = 19) and above-ground biomass (*t* = 7.12, *P*<0.001, n = 12). The LDF was especially distinctive in having virtually all small stems (Fig. 9).

## Discussion

### Comparing Hawaiian Wet and Dry Forests

Examination of how the two Hawaiian forests compared in terms of composition and structure matched and extended the paradigm for differences between mature wet and dry forests described on other tropical islands [32]. As predicted, our results for Hawai‘i are in agreement with comparisons of mature wet and dry forests in Puerto Rico [32]: wet forest had larger diameter trees, greater basal area, and higher biomass than dry forest, and differences between wet and dry forest in tree density, dominance, and species richness were minor (Tables 3–4). Indeed, across many forests, biomass and basal area are typically correlated with climatic variables such as MAT, MAP, and water deficit within and across sites [31], [70], [71], [72], [73], [74], [75]. However, other variables such as substrate age and type [76] are likely to have contributed to structural and floristic differences between LDF and MWF and we cannot ascribe results solely to climate. While both forests occur on young lava, the much higher rainfall of the MWF and variation in substrate type and texture contributed to greater soil development. Lava flow age and substrate type are important determinants of successional stage in Hawai‘i [39]. In addition, differences in disturbance regimes between the two sites may have influenced their forest structure, and their invasive species cover. In the MWF, almost a third of stems were found growing on a substrate other than soil, such as nurse logs and living tree ferns, which likely reflects preferential survivorship on those substrates. Further, canopy dieback of *Metrosideros polymorpha* [45], wind storms [44], and invasive animals [77] may be important factors influencing forest structure. Canopy gaps are larger in the MWF due to the much larger and taller trees that make up the canopy. There is also evidence of pig rooting that may affect seedling regeneration preferences [47], [78], explaining why many stems grow on substrates other than soil. The LDF site is currently fenced from ungulates but the large proportion of multi-stemmed trees and the higher prevalence of regeneration by sprouting suggest adaptation to disturbance [79], [80], [81].

The MWF site distinguishes itself in its abundance and dominance of tree ferns, which form a distinct mid-canopy layer approximately 5 m above the ground. Notably, tree ferns also make up a large proportion of stand basal area or stem density in some temperate rain forests [82], [83], [84] and tropical cloud forests [85], [86], [87], [88] but not in other CTFS sites. In Hawai‘i, tree ferns are common in wet forests at all elevations, and are particularly abundant in areas with more well-developed organic soils as opposed to young lava flows. While the dynamics of tree ferns have not been well studied in tropical environments [89], in Hawaiian forests tree ferns undoubtedly influence forest function, due to their long lifespans, high frond area, slow growth [90], and slow decomposition rates [91]. They also play a critical role as a substrate for tree seedlings [47], [78].

Unlike other CTFS plots that are not heavily impacted by non-native plant species, all forests in Hawai‘i have been invaded to some degree. We purposefully chose sites with low non-native species abundance, but cataloged cover before removal for future long-term studies. Because we are removing the invasive species after data collection, we are not examining the consequences of invasion, but previous work in Hawai‘i has shown that invaders can significantly alter forest functioning [50], [51], [52], [92], [94]. A debate in invasion biology is whether invaders owe their success to their introducing a new function to the community (e.g., N-fixing species) or are simply better competitors [92] and we argue that it is the latter case at our sites. At our sites, invasive grasses were widespread, but, woody invaders are a greater competitive threat (Figs. 7). In the LDF, *Pennisetum setaceum* is widespread, but the vegetation is still dominated by woody species with moderate canopy closure, and reduction of grass cover and fire prevention will reduce its competitive effect in the future. While non-native grasses and herbs are more common than non-native trees across the MWF, their abundance is strongly related to boggy areas, canopy openings, and pig disturbance, and these patches are not likely to expand, but rather to be shaded out in the long term. In MWF, woody invaders such as *Psidium cattleianum* represent much greater threats based on their extreme abundance elsewhere, and traits such as shade tolerance, vegetative reproduction, and animal-dispersed fruits [93], [94]. At present, the MWF has limited cover of woody invaders (Fig. 7), and in that respect is in better condition that the LDF.

Clearly, site-specific properties influence the structure and species composition between the two sites, but our study also highlights that at the island scale (1 million ha), climate likely exerts a strong influence, both directly and indirectly [67]. These differences matched patterns found in continental forests, where diversity measures as well as structural measures correlate negatively with the length or severity of the dry season [68], [69].

### Structure and Diversity across Tropical Forests Globally

The Hawaiian forest data allowed for the examination of the question of how forest structure varies across species diversity gradients across a much wider range of tree species diversity than was previously available. One of the most striking conclusions of our study is that, despite the extremely low species richness of the Hawai‘i FDPs, some structural variables, particularly those for the wet forest, were well within the range of values for the world's most diverse tropical forests (Table 2). For example, stem densities for Hawaiian MWF and LDF were similar to those of the hyper-species-rich Yasun??? FDP (Fig. 8), while biomass and basal area of the Hawai‘i MWF (excluding tree ferns) were similar to those of the higher diversity forests in the CTFS network (Fig. 9). The inclusion of tree ferns increased basal area values by 52%, but only increased biomass by 11% (Tables 3 and 4). Notably, the LDF had among the lowest basal area and biomass in the CTFS network, consistent with this site having the lowest precipitation of all FDPs (Table 2). It should also be noted that the LDF is dry year-round, while other dry sites in the CTFS network are seasonally dry (Table 2).

The low floristic richness and population structure of the Hawaiian forest plots represented strong convergence with other island forests. Hawaiian forests had fewer species per family and greater average population densities for each species, as seen in other very isolated sites [95]. High relative dominance values were consistent with island forests having greater dominance by the most common family than mainland tropical forests (Table 2). In the MWF and LDF, 37% and 74% of basal area respectively were accounted for by a single canopy dominant species. On average 20% of species were rare in Hawaiian forests (defined as ≤1 tree/ha), by contrast with 42% on average across other high-diversity forests [96]. It is likely that the patterns of high basal area dominance in Hawaiian forests arose due to the biogeographic consequences of isolation, but we cannot rule out species loss due to human disturbance and invasive species of multiple trophic levels [97], [98], [99], [100], [101].

In conclusion, Hawaiian forests have among the lowest species richness and highest endemism rates globally, but in a number of key structural variables both of these forests were similar to even the highest diversity tropical forests in the CTFS network. Future work could examine the evolutionary consequences of such a limited species pool. Biodiversity theory developed in high-diversity tropical forests emphasizes that competitive interactions among species are unlikely on evolutionary time scales because any given two species are rarely consistent neighbors [9]. However, in low-diversity forest any two given species have far greater potential for competitive interactions than in high-diversity tropical forests [102], [103]. The addition of Hawai‘i to the global plot network enables investigations of the consequences of such differences across a very wide range in species diversity and environmental gradients.

## Supporting Information

File S1 **Methods S1.** Detailed methods and description of situations where field site conditions dictated a different or entirely new methodology by adopted than standardized CTFS protocol in [1].
(DOCX)

File S2 **Supporting tables.** **Table S1**. Values and equations used for estimating aboveground biomass (AGB) in the montane wet forest (MWF) and lowland dry forest sites (LDF). **Table S2**. Species ≥1 cm diameter at breast height recorded in Laupāhoehoe (montane wet forest) plot with canopy dominants in bold. **Table S3**. Percentage of individuals in the Laupāhoehoe (montane wet forest) plot growing on each substrate type. **Table S4**. Size and multiple stem characteristics of the species species in Laupāhoehoe (montane wet forest) and Pālamanui (lowland dry forest) plots; species abbreviations as in Table S4. **Table S5**. References from Table 1.
(DOCX)
