# Supplementary material for: Forest Structure in Low-Diversity Tropical Forests: A Study of Hawaiian Wet and Dry Forests
Source: PLoS One. 2014 Aug 27;9(8):e103268. doi: 10.1371/journal.pone.0103268 (PMC4146472; doi:10.1371/journal.pone.0103268)
Supplement: File S1 — Methods S1. Detailed methods and description of situations where field site conditions dictated a different or entirely new methodology by adopted than standardized CTFS protocol in [1]. (DOCX) [file pone.0103268.s001.docx]

**Methods S1.** Detailed methods and description of situations where field site conditions dictated a different or entirely new methodology by adopted than standardized CTFS protocol in [1].

Each 4 ha plot was subdivided into 20 m × 20 m quadrats and further subdivided into 5 m × 5 m subquadrats for tree mapping. Quadrat corners were located from an initial reference point using a TOPCON Electronic Total Station GTS-210 Series (Livermore, CA) for the LDF, and a Laser Rangefinder and MapStar Angle Encoder (Laser Technology, Inc., Centennial, CO) for the MWF and quadrat posts were surveyed to within ± 20 cm and ± 0.5° accuracy. The 5 m × 5 m subquadrats were established with transect tapes, and thus have a lower precision.

We took our data electronically, which differs from other FDPs; for tree mapping and field measurements, we customized a mapping program on handheld field computers (ArcPad Version 7.0.1 Allegro CX Field PCs; Juniper Systems, Inc., Logan, UT; [2]. As in other CTFS FDPs, exceptions to the protocol were necessary due to unique growth conditions and tree morphology. The MWF contained numerous large *M.* *polymorpha* trees that were difficult to measure at 130 cm due to buttresses, tree clusters, or epiphytes (199 trees; Fig. 1). When trees of different species were grafted together, each tree was tagged as a separate individual. We used ladders or tree climbing equipment to measure trunk diameters at a height where the main stem was free from obstructions and protrusions. To estimate diameter at 130 cm for *M.* *polymorpha* trees measured above 2 m height, we developed a tapering equation from nine randomly selected *M.* *polymorpha* trees of clean form. We measured diameter every 1 m from 2 m above the tree base to up to 10 m tree height. Assuming approximately even tapering along the stem, the narrowing of the stem from 130 cm height to the measurement height would be proportional to measurement height. We fitted the data by the equation:

*Diameter at height x / diameter at 130 cm height = a × height x + b* (1).

The regression was strongly significant, with *a* = -0.0006 and *b* = 1.08 (*r*^2^ = 0.68, *n* = 67; *P*< 0.001). We used this equation to estimate dbh at 130 cm height for *M.* *polymorpha* that were originally measured above 2 m height. We used these estimated dbh values for forest structure calculations in this paper.

At the MWF, tree ferns are exceptionally abundant. Despite their importance in the understory, there are many tree ferns that are as tall as understory tree species but do not have trunks that reach 130 cm height. Because of their abundance, we included tree ferns with trunks that reached 50 cm height and measured the diameter at 50 cm height, rather than the 130 cm used as cut off for dicotyledonous trees. However, to allow comparisons with other FDPs, the calculations of structural variables for this paper were based on the estimated number of tree ferns that we expected would reach 130 cm height based on the proportion of a random sample of 435 trees ferns that reached 130 cm height. Specifically, we randomly selected 200 *Cibotium glaucum* and 200 *C. menziesii* (only 196 *C. menziesii* were refound and remeasured) and measured the height of each tree fern from the base to where the fronds separate from the trunk. For the third, less common, tree fern species, *C. chamissoi*, we were able to measure all individuals. For this species, we excluded individuals that did not reach 130 cm height.

To determine if the diameter of tree ferns measured at 50 cm height was equivalent to the diameter at 130 cm height, we measured the diameters of arandomly selected subset of tree ferns at both 50 cm and 130 cm height (for individuals that reached 130 cm height). Diameters at the two heights were not different for *C. glaucum* and *C. menziesii* (paired t-tests, *P*> 0.05), and thus we used the diameter at 50 cm as the dbh measurement. For *C. chamissoi*, we conducted calculations with the dbh measured at the standard height for all individuals that reached 130 cm height.

We conducted a number of additional forest structure measurements beyond tree size to gain a better understanding of potential linkages with diversity. In the MWF, many trees and shrubs grow epiphytically or establish on nurse logs, and we recorded the rooting substrate for each tree within categories: soil, live tree fern, dead tree fern, live tree, rock or root mats. We also estimated and mapped the non-native cover by herbaceous, shrub and tree species. The MWF sub-quadrats averaged 5-25% weed cover, most of which was herbaceous, whereas the LDF sub-quadrats averaged 25-50% cover, mainly woody species. In the MWF there were only a few stems of non-native trees > 5 cm in dbh: one large *Fraxinus uhdei* (32.3 cm) and one large *Psidium cattleianum* (6.2 cm). In the LDF, there were 27 *Grevillea robusta* trees > 5 cm in dbh (mean ± SE of 20.0 ± 8.2 cm), 7 *Leucana leucocephalum* trees (5.9 ± 0.8 cm) and 20 *Schinus terebinthius* trees (11.0 ± 1.5 cm). After measurement the non-native trees were girdled and sprayed with herbicide.

1. Condit R (1998) Tropical forest census plots. Berlin: Springer-Verlag, Berlin, Germany.

2. Inman-Narahari F, Giardina C, Ostertag R, Cordell S, Sack L (2010) Digital data collection in forest dynamics plots. Methods in Ecology and Evolution 1: 274-279.
