# Supplementary material for: Forest Structure in Low-Diversity Tropical Forests: A Study of Hawaiian Wet and Dry Forests
Source: PLoS One. 2014 Aug 27;9(8):e103268. doi: 10.1371/journal.pone.0103268 (PMC4146472; doi:10.1371/journal.pone.0103268)
Supplement: File S2 — Supporting tables. Table S1. Values and equations used for estimating aboveground biomass (AGB) in the montane wet forest (MWF) and lowland dry forest sites (LDF). Table S2. Species ≥1 cm diameter at breast height recorded in Laupāhoehoe (montane wet forest) plot with canopy dominants in bold. Table S3. Percentage of individuals in the Laupāhoehoe (montane wet forest) plot growing on each substrate type. Table S4. Size and multiple stem characteristics of the species species in Laupāhoehoe (montane wet forest) and Pālamanui (lowland dry forest) plots; species abbreviations as in Table S4. Table S5. References from Table 1. (DOCX) [file pone.0103268.s002.docx]

**Table S1.**

| **Laupāhoehoe montane wet forest** | | | | | | |
| --- | --- | --- | --- | --- | --- | --- |
| **Species** | **ρ** | **ρ source** | **Height model** | **Height model source** | **Biomass equation** | **Biomass equation source** |
| ACAKOA | 0.550 | S¹ | exp(0.1795 + 1.0160 × ln(D)-0.0800 × ln(D)²) × 1.0156 | S¹ | exp(-2.3270+2.3500 × ln(D)) × 1.0171 | S¹ if D ≤30; otherwise GWF² |
| ANTPLA | 0.670 | S¹ | 1.1240 + 8.5503 × (1-exp(-0.1079 × D)) | S¹ | exp(-2.557 + 0.940 × ln(ρD^²^H)) | GWF² |
| BROARG | 0.360 | S^3^ | exp(0.5120 + 0.7583 × ln(D)-0.0322 × ln(D)^²^) × 1.0409 | GWF¹ | exp(-2.557 + 0.940 × ln(ρD^²^H)) | GWF² |
| CHETRI | 0.470 | S¹ | 12.6477 × (1-exp(-0.1365 × D)) | S¹ | exp(-2.557 + 0.940 × ln(ρD^²^H)) | GWF² |
| CIBCHA | 0.210 | S¹ | exp(0.6457 + 1.5932 × ln(D)) × 1.2763/100 | S¹ | π × (D/2)² × H × 100 × ρ/1000 | Genus specific¹ |
| CIBGLA | 0.220 | S¹ | exp(-0.6277 + 1.6910 × ln(D)) × 1.1386/100 | S¹ | π × (D/2)² × H × 100 × ρ/1000 | Genus specific¹ |
| CIBMEN | 0.190 | S¹ | exp(-0.6549 + 1.8683 × ln(D)) × 1.1705/100 | S¹ | π × (D/2)² × H × 100 × ρ/1000 | Genus specific¹ |
| CLEPAR | 0.500 | Default value¹ | exp(0.5120 + 0.7583 × ln(D)-0.0322 × ln(D)^²^) × 1.0409 | GWF¹ | exp(-2.557 + 0.940 × ln(ρD^²^H)) | GWF² |
| COPRHY | 0.480 | S¹ | 10.2252 × (1-exp(-0.2257 × D)) | S¹ | exp(-2.557 + 0.940 × ln(ρD^²^H)) | GWF² |
| HEDHIL | 0.380 | S¹ | exp(0.5120 + 0.7583 × ln(D)-0.0322 × ln(D)^²^) × 1.0409 | GWF¹ | exp(-2.557 + 0.940 × ln(ρD^²^H)) | GWF² |
| ILEANO | 0.480 | S¹ | 13.0821 × (1-exp(-0.1339 × D)) | S¹ | exp(-2.557 + 0.940 × ln(ρD^²^H)) | GWF² |
| LEPTAM | 0.670 | S^3^ | exp(0.5120 + 0.7583 × ln(D)-0.0322 × ln(D)^²^) × 1.0409 | GWF¹ | exp(-2.557 + 0.940 × ln(ρD^²^H)) | GWF² |
| MELCLU | 0.480 | Genus average⁴ | exp(0.5120 + 0.7583 × ln(D)-0.0322 × ln(D)^²^) × 1.0409 | GWF¹ | exp(-2.557 + 0.940 × ln(ρD^²^H)) | GWF² |
| METPOL | 0.690 | S¹ | 5.6702 × ln(D) - 4.0406 | S¹ | exp(-2.1311+2.5011 × ln(D)) × 1.0671 | S¹ if D ≤33, otherwise GWF² |
| MYRLES | 0.500 | S^3^ | exp(0.5120 + 0.7583 × ln(D)-0.0322 × ln(D)^²^) × 1.0409 | GWF¹ | exp(-2.557 + 0.940 × ln(ρD^²^H)) | GWF² |
| MYRSAN | 0.610 | S^3^ | 2.1002 + 8.1291 × (1-exp(-0.0489 × D)) | S¹ | exp(-2.557 + 0.940 × ln(ρD^²^H)) | GWF² |
| PERSAN | 0.410 | S¹ | 1.3360 + 11.8919 × (1-exp(-0.0772 × D)) | S¹ | exp(-2.557 + 0.940 × ln(ρD^²^H)) | GWF² |
| PIPALB | 0.300 | S¹ | exp(0.5120 + 0.7583 × ln(D)-0.0322 × ln(D)^²^) × 1.0409 | GWF¹ | exp(-2.557 + 0.940 × ln(ρD^²^H)) | GWF² |
| PSYHAW | 0.540 | S^3^ | exp(0.5120 + 0.7583 × ln(D)-0.0322 × ln(D)^²^) × 1.0409 | GWF¹ | exp(-2.557 + 0.940 × ln(ρD^²^H)) | GWF² |
| TREGRA | 0.500 | S¹ | exp(0.5120 + 0.7583 × ln(D)-0.0322 × ln(D)^²^) × 1.0409 | GWF¹ | exp(-2.557 + 0.940 × ln(ρD^²^H)) | GWF² |
| VACCAL | 0.500 | S¹ | exp(0.5120 + 0.7583 × ln(D)-0.0322 × ln(D)^²^) × 1.0409 | GWF¹ | exp(-2.557 + 0.940 × ln(ρD^²^H)) | GWF² |

| **Pālamanui lowland dry forest** | | | | | | |
| --- | --- | --- | --- | --- | --- | --- |
| **Species** | **ρ** | **ρ source** | **Height model** | **Height model source** | **Biomass equation** | **Biomass equation source** |
| DIOSAN | 0.740 | S¹ | 6.0846 × (1-exp(-0.1010 × D)) | S¹ | 0.115 × D × exp(2.45) | S^4^ and GDF² |
| DODVIS | 0.947 | S^5^ | exp(0.3480 + 0.6056 × ln(D)-0.0246 × ln(D)^2^) × 1.0514 | GDF¹ | exp(-2.187+0.916 × ln(ρD²H)) | GDF² |
| ERYSAN | 0.290 | S^5^ | exp(0.3480 + 0.6056 × ln(D)-0.0246 × ln(D)^2^) × 1.0514 | GDF¹ | exp(-2.187+0.916 × ln(ρD²H)) | GDF² |
| EUPMUL | 0.470 | Closely related genus (*Euphorbia*)^5^ | exp(0.3480 + 0.6056 × ln(D)-0.0246 × ln(D)^2^) × 1.0514 | GDF¹ | exp(-2.187+0.916 × ln(ρD²H)) | GDF² |
| METPOL | 0.690 | S¹ | 14.1340 × (1-exp(-0.0573 × D)) | S¹ | exp(-2.1311+2.5011x ln(D)) × 1.0671) | S¹ if D ≤33, otherwise GDF ² |
| MYOSAN | 0.881 | Genus average^5^ | exp(0.3480 + 0.6056 × ln(D)-0.0246 × ln(D)^2^) × 1.0514 | GDF¹ | exp(-2.187+0.916 × ln(ρD²H)) | GDF² |
| OSTANT | 0.700 | Closely related genera within family^5^ | exp(0.3480 + 0.6056 × ln(D)-0.0246 × ln(D)^2^) × 1.0514 | GDF¹ | exp(-2.187+0.916 × ln(ρD²H)) | GDF² |
| PITTER | 0.670 | Genus average^5^ | exp(0.3480 + 0.6056 × ln(D)-0.0246 × ln(D)^2^) × 1.0514 | GDF¹ | exp(-2.187+0.916 × ln(ρD²H)) | GDF² |
| PLEHAW | 0.418 | Closely related genus (*Dracaena*) ^5^ | exp(0.3480 + 0.6056 × ln(D)-0.0246 × ln(D)^2^) × 1.0514 | GDF¹ | exp(-2.187+0.916 × ln(ρD²H)) | GDF² |
| PSYODO | 0.870 | Genus average^5^ | 6.5422 × (1-exp(-0.1832 × D)) | S¹ | 0.205 × D × exp(2.221) | S^4^ and GDF² |
| SANPAN | 0.760 | Genus average^5^ | exp(0.4386 + 0.3883 × ln(D)) × 1.0265 | S¹ | exp(-2.187+0.916 × ln(ρD²H)) | GDF² |
| SENGAU | 0.600 | Genus average^5^ | exp(0.3480 + 0.6056 × ln(D)-0.0246 × ln(D)^2^) × 1.0514 | GDF¹ | exp(-2.187+0.916 × ln(ρD²H)) | GDF² |
| SIDFAL | 0.452 | Closely related genera within family^5^ | exp(0.3480 + 0.6056 × ln(D)-0.0246 × ln(D)^2^) × 1.0514 | GDF¹ | exp(-2.187+0.916 × ln(ρD²H)) | GDF² |
| SOPCHR | 0.640 | Genus average^5^ | 5.3775 × (1-exp(-0.2262 × D)) | S¹ | exp(-2.187+0.916 × ln(ρD²H)) | GDF² |
| WIKSAN | 0.425 | Closely related genera within family^5^ | exp(0.3480 + 0.6056 × ln(D)-0.0246 × ln(D)^2^) × 1.0514 | GDF¹ | exp(-2.187+0.916 × ln(ρD²H)) | GDF² |
|  |  |  |  |  |  |  |

We used biomass equations employing wood density and tree height; tree height was derived from diameter at breast height as in Asner et. al (2011) as further detailed in Methods (D = tree diameter, ρ = wood specific gravity, H = tree height); species abbreviations as in Appendix B. S = species-specific; GWF = general wet forest; GDF = general dry forest.

^1^ Asner GP, Hughes RF, Mascaro J, Uowolo AL, Knapp DE, et al. (2011) High-resolution carbon mapping on the million-hectare island of Hawai‘i. Frontiers in Ecology and the Environment 9: 434-439.

^2^ Chave J, Andalo C, Brown S, Cairns MA, Chambers JQ, et al. (2005) Tree allometry and improved estimation of carbon stocks and balance in tropical forests. Oecologia 145: 87-99.

³ Litton and Giardina, unpub.

^4^ Litton CM, Sandquist DR, Cordell S (2006) Effects of non-native grass invasion on aboveground carbon pools and tree population structure in a tropical dry forest of Hawaii. Forest Ecology and Management 231: 105-113.

^5^ Zanne AE, Lopez-Gonzalez G, Coomes DA, Ilic J, Jansen S, et al. (2009) Towards a worldwide wood economics spectrum. Dryad Digital Repository doi: 10.5061/dryad.5234.

**Table S2.**

**Laupāhoehoe montane wet forest**

| **Family** | **Species** | **Abbreviation** | **Common name** | **Origin** | **Life form** |
| --- | --- | --- | --- | --- | --- |
| Fabaceae | *Acacia koa* A. Gray | **ACAKOA** | koa | E | Tree |
| Phyllanthaceae | *Antidesma platyphyllum* H. Mann | ANTPLA | hame | E | Tree |
| Hydrangeaceae | *Broussaisia arguta* Gaudich. | BROARG | kanawao | E | Shrub |
| Araliaceae | *Cheirodendron trigynum* (Gaudich.) A. Heller | CHETRI | ‘ōlapa | E | Tree |
| Cibotiaceae | *Cibotium chamissoi* Kaulf. | CIBCHA | hāpu‘u | E | Tree fern |
| Cibotiaceae | *Cibotium glaucum* (Sm.) Hook. & Arn. | CIBGLA | hāpu‘u pulu | E | Tree fern |
| Cibotiaceae | *Cibotium menziesii* Hook. | CIBMEN | hāpu‘u ‘i‘i | E | Tree fern |
| Campanulaceae | *Clermontia parviflora* Gaudich. ex A. Gray | CLEPAR | ‘ōhā kē pau | E | Shrub |
| Rubiaceae | *Coprosma rhynchocarpa* A. Gray | COPRYH | pilo | E | Tree |
| Rubiaceae | *Hedyotis hillebrandii* (Fosberg) W. L. Wagner & D. R. Herbst | HEDHIL | manono | E | Shrub |
| Aquifoliaceae | *Ilex anomala* Hook. & Arn. | ILEANO | kāwa‘u | E | Tree |
| Ericaceae | *Leptecophylla tameiameiae* (Cham. & Schltdl.) C. M. Weiller | LEPTAM | pūkiawe | I | Shrub |
| Rutaceae | *Melicope clusiaefolia* (A. Gray) T. G. Hartley & B. C. Stone | MELCLU | alani | E | Tree |
| Myrtaceae | *Metrosideros polymorpha* (H. Lév.) H. St. John | **METPOL** | ‘ōhi‘a lehua | E | Tree |
| Myrsinaceae | *Myrsine lessertiana* A. DC. | MYRLES | kōlea lau nui | E | Tree |
| Myrsinaceae | *Myrsine sandwicensis* A. DC. | MYRSAN | kōlea lau li‘i | E | Tree |
| Celastraceae | *Perrottetia sandwicensis* A. Gray | PERSAN | olomea | E | Tree |
| Urticaceae | *Pipturus albidus* (Hook. & Arn.) A. Gray | PIPALB | māmaki | E | Shrub |
| Rubiaceae | *Psychotria hawaiiensis* (A. Gray) Fosberg | PSYHAW | kōpiko | E | Tree |
| Campanulaceae | *Trematolobelia grandiflora* (Rock) O. Deg. | TREGRA | koli‘i | E | Shrub |
| Ericaceae | *Vaccinium calycinum* Sm. | VACCAL | ‘ōhelo kau lā‘au | E | Shrub |

**Pālamanui lowland dry forest**

| **Family** | **Species** | **Abbreviation** | **Common name** | **Origin** | **Life form** |
| --- | --- | --- | --- | --- | --- |
| Ebenaceae | *Diospyros sandwicensis* (A. DC.) Fosberg | **DIOSAN** | lama | E | Tree |
| Sapindaceae | *Dodonaea viscosa* Jacq. | DODVIS | ‘a‘ali‘i | I | Shrub |
| Fabaceae | *Erythrina sandwicensis* O. Deg. | ERYSAN | wiliwili | E | Tree |
| Euphorbiaceae | *Euphorbia multiformis* (Hook. & Arn.) Croizat & O. Deg., (Boiss.) O. Deg. & I. Deg. | EUPMUL | ‘akoko | E | Shrub |
| Myrtaceae | *Metrosideros polymorpha* Gaudich. | METPOL | ‘ōhi‘a lehua | E | Tree |
| Scrophulariaceae | *Myoporum sandwicense* A. Gray | MYOSAN | naio | I | Tree |
| Rosaceae | *Osteomeles anthyllidifolia* (Sm.) Lindl. | OSTANT | ‘ūlei | I | Shrub |
| Pittosporaceae | *Pittosporum terminalioides* Planch. ex A. Gray | PITTER | hō‘awa | E | Tree |
| Asparagaceae | *Pleomele hawaiiensis* O. Deg. & I. Deg. | PLEHAW | hala pepe | E | Tree |
| Rubiaceae | *Psydrax odoratum* (G. Forst.) A. C. Sm. & S. P. Darwin | PSYODO | alahe‘e | I | Tree |
| Santalaceae | *Santalum paniculatum* Hook. & Arn. | SANPAN | ‘iliahi | E | Tree |
| Fabaceae | *Senna gaudichaudii* (Hook. & Arn.) H. S. Irwin & Barneby | SENGAU | kolomona | I | Tree |
| Malvaceae | *Sida fallax* Walp. | SIDFAL | ‘ilima | I | Shrub |
| Fabaceae | *Sophora chrysophylla* (Salisb.) Seem. | SOPCHR | māmane | E | Tree |
| Thymelaeaceae | *Wikstroemia sandwicensis* Meisn. | WIKSAN | ‘ākia | E | Tree |

E: endemic, I: indigenous; nomenclature follows Wagner et al. (1999, and online updates (http://botany.si.edu/pacificislandbiodiversity/hawaiianflora/index.htm) and the Angiosperm Phylogeny Website (www.mobot.org/mobot/research/apweb/); websites accessed August 2011

Reference:

Wagner WL, Herbst DR (1999) Manual of the Flowering Plants of Hawai‘i. Honolulu: Bishop Museum.

**Table S3.**

|  | **Dead tree fern** | **Live tree** | **Live tree fern** | **Log** | **Root mat** | **Rock** | **Soil** | **% Raised** |
| --- | --- | --- | --- | --- | --- | --- | --- | --- |
|  |  |  |  |  |  |  |  |  |
| ACAKOA | 2.11 | 0 | 0 | 0.704 | 17.6 | 78.9 | 0.704 | 2.82 |
| BROARG | 11.4 | 0 | 2.58 | 0.738 | 31.0 | 3.69 | 50.2 | 14.8 |
| CHETRI | 24.9 | 4.37 | 9.19 | 8.37 | 29.4 | 1.05 | 22.7 | 46.9 |
| CIBCHA | 11.8 | 0 | 0 | 0 | 20.6 | 67.6 | 0 | 11.8 |
| CIBGLA | 3.83 | 0 | 0.616 | 0.79 | 24.5 | 0.748 | 69.1 | 5.23 |
| CIBMEN | 13.9 | 1.21 | 1.40 | 6.42 | 29.6 | 0 | 47.5 | 22.9 |
| COPRHY | 3.50 | 0 | 1.85 | 1.54 | 25.8 | 6.38 | 60.8 | 6.89 |
| HEDHIL | 2.27 | 0 | 0 | 0 | 34.1 | 13.6 | 50.0 | 2.27 |
| ILEANO | 33.1 | 2.69 | 6.01 | 9.53 | 25.0 | 0.104 | 23.6 | 51.3 |
| METPOL | 18.3 | 1.56 | 6.54 | 9.73 | 33.8 | 1.44 | 28.6 | 36.2 |
| MYRLES | 26.2 | 2.95 | 5.06 | 11.8 | 34.2 | 0.422 | 19.4 | 46.0 |
| VACCAL | 25.1 | 3.14 | 14.1 | 4.31 | 38.8 |  | 14.5 | 46.7 |
|  |  |  |  |  |  |  |  |  |
| **All Species** | **16.9** | **2.06** | **5.30** | **6.31** | **28.9** | **1.45** | **39.1** | **30.5** |

Only species with 40 or more individuals are listed; all species refers to a total of 12,310 stems for which we have substrate data; the stems growing on live or dead tree ferns, trees, or logs were considered raised off of the ground.

**Table S4.**

**Laupāhoehoe montane wet forest**

| **Species** | **Mean dbh (cm)** | **Minimum dbh (cm)** | **Maximum dbh (cm)** | **Mean no. of stems/Individual** | **Maximum no. of stems/Individual** |
| --- | --- | --- | --- | --- | --- |
| ACAKOA | 38.6 | 1.06 | 113 | 1.02 | 2 |
| ANTPLA | 2.80 | 2.80 | 2.80 | 1.00 | 1 |
| BROARG | 2.01 | 1.00 | 4.82 | 2.28 | 10 |
| CHETRI | 6.05 | 1.00 | 91.5 | 1.29 | 9 |
| CIBCHA | 16.9 | 8.30 | 36.5 | 1.09 | 3 |
| CIBGLA | 19.0 | 6.10 | 56.2 | 1.03 | 4 |
| CIBMEN | 23.0 | 7.90 | 72.8 | 1.03 | 4 |
| CLEPAR | 2.05 | 1.05 | 4.20 | 1.37 | 3 |
| COPRHY | 4.59 | 1.00 | 15.4 | 1.18 | 5 |
| HEDHIL | 4.20 | 1.01 | 29.5 | 1.57 | 4 |
| ILEANO | 3.34 | 1.00 | 29.2 | 1.45 | 12 |
| LEPTAM | 1.61 | 1.21 | 2.00 | 1.00 | 1 |
| MELCLU | 2.54 | 1.01 | 4.40 | 1.31 | 2 |
| METPOL | 10.6 | 1.00 | 154 | 1.29 | 16 |
| MYRLES | 2.73 | 1.00 | 10.6 | 1.46 | 10 |
| MYRSAN | 2.88 | 1.00 | 7.40 | 1.00 | 1 |
| PERSAN | 2.36 | 1.03 | 7.80 | 1.80 | 9 |
| PIPALB | 4.66 | 3.40 | 6.30 | 2.25 | 4 |
| PSYHAW | 2.63 | 1.11 | 8.20 | 1.30 | 2 |
| TREGRA | 1.31 | 1.12 | 1.49 | 1.00 | 1 |
| VACCAL | 1.61 | 1.00 | 4.13 | 2.80 | 18 |

**Pālamanui lowland dry forest**

| **Species** | **Mean dbh (cm)** | **Minimum dbh (cm)** | **Maximum dbh (cm)** | **Mean no. of stems/Individual** | **Maximum no. of stems/Individual** |
| --- | --- | --- | --- | --- | --- |
| DIOSAN | 7.20 | 1.00 | 34.3 | 2.48 | 19 |
| DODVIS | 1.70 | 1.00 | 6.00 | 2.48 | 17 |
| ERYSAN | 4.66 | 4.66 | 4.66 | 4.00 | 4 |
| EUPMUL | 2.42 | 1.02 | 5.80 | 1.91 | 6 |
| METPOL | 38.18 | 26.00 | 53.1 | 3.40 | 5 |
| MYOSAN | 4.04 | 1.40 | 7.40 | 5.75 | 11 |
| OSTANT | 2.55 | 1.00 | 5.90 | 7.61 | 32 |
| PITTER | 4.53 | 4.53 | 4.53 | 8.00 | 8 |
| PLEHAW | 3.28 | 1.76 | 4.80 | 1.00 | 1 |
| PSYODO | 1.70 | 1.00 | 9.05 | 2.22 | 30 |
| SANPAN | 4.09 | 1.08 | 11.7 | 2.00 | 17 |
| SENGAU | 2.26 | 1.00 | 4.19 | 1.33 | 3 |
| SIDFAL | 1.54 | 1.02 | 3.62 | 1.00 | 1 |
| SOPCHR | 2.34 | 1.00 | 6.20 | 1.65 | 9 |
| WIKSAN | 3.32 | 1.00 | 8.20 | 2.91 | 17 |

**Table S5.**

^1^ Juvik SP, Juvik JO, Paradise TR (1998) Atlas of Hawaii, 3rd edition. Honolulu, HI: University of Hawaii Press.

^2^ Vitousek PM (2004) Nutrient cycling and limitation: Hawai`i as a model system. Princeton, N.J: Princeton University Press.

^3^ Burton PJ (1980) Light regimes and *Metrosideros* regeneration in a Hawaiian montane rain forest. M.S. thesis. Honolulu: University of Hawaii at Manoa.

^4^ Burton PJ, Mueller-Dombois D (1984) Response of *Metrosideros polymorpha* seedlings to experimental canopy opening. Ecology 65: 779-791.

^5^ Pattison RR, Goldstein, G., and Ares, A (1998) Growth, biomass allocation and photosynthesis of invasive and native Hawaiian rainforest species. Oecologia 117: 449-459.

^6^ Scowcroft PG, Jeffrey J (1999) Potential significance of frost, topographic relief, and *Acacia koa* stands to restoration of mesic Hawaiian forests on abandoned rangeland. Forest Ecology and Management 114: 447-458.

^7^ Cordell S, Sandquist DR, C. Litton, Cabin RJ, Thaxton J, et al. An invasive grass has significant impacts on tropical dry forest ecosystems in Hawaii: The role of science in landscape level resource management and native forest restoration in West Hawaii; 2004; Victoria, Canada.

^8^ Denslow JS, A. L. Uowolo, Hughes RF (2006) Limitations to seedling establishment in a mesic Hawaiian forest. Oecologia 148: 118-128.

^9^ McDaniel S, Ostertag R (2010) Strategic light manipulation as a restoration strategy to reduce alien grasses and encourage native regeneration in Hawaiian mesic forests. Applied Vegetation Science 13: 280-290.

^10^ Wagner WL, Herbst DR (1999) Manual of the Flowering Plants of Hawai‘i. Honolulu: Bishop Museum.

^11^ Myers N, Mittermeier RA, Mittermeier CG, da Fonseca GAB, Kent J (2000) Biodiversity hotspots for conservation priorities. Nature 403: 853-858.

^12^ Vitousek PM, Aplet G, Turner D, Lockwood JJ (1992) The Mauna Loa environmental matrix: foliar and soil nutrients. Oecologia 89: 372-382.

^13^ Cornwell WK, Bhaskar R, Sack L, Cordell S, Lunch CK (2007) Adjustment of structure and function of Hawaiian *Metrosideros polymorpha* at high vs. low precipitation. Functional Ecology 21: 1063-1071.

^14^ Hoof J, Sack L, Webb DT, Nilsen ET (2008) Contrasting structure and function of pubescent and glabrous varieties of Hawaiian *Metrosideros polymorpha* (Myrtaceae) at high elevation. Biotropica 40: 113-118.

^15^ Mueller-Dombois D (1987) Forest dynamics in Hawaii. Trends in Ecology and Evolution 2: 216-220.

^16^ Kitayama K, Mueller-Dombois D (1995) Biological invasion on an oceanic island mountain: do alien plant species have wider ecological ranges than native species? Journal of Vegetation Science 6: 667-674.

^17^ Drake DR (1993) Germination requirements of *Metrosideros polymorpha*, the dominant tree of Hawaiian lava flows and rain forests. Biotropica 25: 461-467.

^18^ Gerrish G, Mueller-Dombois D (1999) Measuring stem growth rates for determining age and cohort analysis of a tropical evergreen tree. Pacific Science 53: 418-429.

^19^ Hart PJ (2010) Tree growth and age in an ancient Hawaiian wet forest: vegetation dynamics at two spatial scales. Journal of Tropical Ecology 26: 1-11.

^20^ Ostertag R, Cordell S, Michaud J, Cole TC, Schulten JR, et al. (2009) Ecosystem and restoration consequences of invasive woody species removal in Hawaiian lowland wet forest. Ecosystems 12: 503-515.

^21^ Scowcroft PG (1992) Role of decaying logs and other organic seedbeds in natural regeneration of Hawaiian forest species on abandoned montane pasture. USDA Forest Service Gen Tech Rep PSW-129: 67-73.

^22^ Kellner JR, Asner GP (2009) Convergent structural responses of tropical forests to diverse disturbance regimes. Ecology Letters 12: 1-11..

^23^ Price JP, Wagner WL (2004) Speciation in Hawaiian angiosperm lineages: cause, consequence, and mode. Evolution 58: 2185-2200.

^24^ Ziegler AC (2002) Hawaiian natural history, ecology, and evolution. Honolulu, HI: University of Hawaii Press.

^25^ Shiels AB (2011) Frugivory by introduced black rats (*Rattus rattus*) promotes dispersal of invasive plant seeds. Biological Invasions 13: 781-792.

^26^ Funk JL, Throop HL (2010) Enemy release and plant invasion: patterns of defensive traits and leaf damage in Hawaii. Oecologia 162: 815-823.

^27^ Janzen DH (1971) Seed predation by animals. Annual Review of Ecology and Systematics 2: 465-492.

^28^ Foster JT, Robinson SK (2007) Introduced birds and the fate of Hawaiian rainforests. Conservation Biology 21: 1248-1257.
